# Lidocaine and bupivacaine as part of multimodal pain management in a C57BL/6J laparotomy mouse model

**DOI:** 10.1038/s41598-021-90331-2

**Published:** 2021-05-25

**Authors:** Mattea S. Durst, Margarete Arras, Rupert Palme, Steven R. Talbot, Paulin Jirkof

**Affiliations:** 1 Centre for Surgical Research, University Hospital Zurich, University of Zurich, Sternwartstrasse 6, 8091 Zurich, Switzerland; 2 Unit of Physiology, Pathophysiology and Experimental Endocrinology, Department of Biomedical Sciences, University of Veterinary Medicine, Vienna, Austria; 3 Institute for Laboratory Animal Science, Hannover Medical School, Hannover, Germany; 4 Office of Animal Welfare and 3R, University of Zurich, Zurich, Switzerland

**Keywords:** Behavioural methods, Mouse, Pain management, Surgery

## Abstract

While the use of local anesthesia as part of multimodal pain management is common practice in human and veterinarian surgery, these drugs are not applied routinely in rodent surgery. Several recommendations on the use of local anesthesia exist, but systematic studies on their efficacy and side effects are lacking. In the present study, male and female C57BL/6J mice were subjected to a sham vasectomy or a sham embryo transfer, respectively. We tested whether a mixture of subcutaneously injected Lidocaine and Bupivacaine in combination with systemic Paracetamol applied via drinking water results in superior pain relief when compared to treatment with local anesthesia or Paracetamol alone. We applied a combination of methods to assess behavioral, emotional, and physiological changes indicative of pain. Voluntary Paracetamol intake via drinking water reached the target dosage of 200 mg/kg in most animals. Local anesthesia did not lead to obvious side effects such as irregular wound healing or systemic disorders. No relevant sex differences were detected in our study. Sevoflurane anesthesia and surgery affected physiological and behavioral measurements. Surprisingly, Paracetamol treatment alone significantly increased the Mouse Grimace Scale. Taken together, mice treated with a combination of local anesthesia and systemic analgesia did not show fewer signs of post-surgical pain or improved recovery compared to animals treated with either local anesthesia or Paracetamol.

## Introduction

Ethical, legal, and scientific reasons necessitate the avoidance or minimization of pain when researchers are conducting animal experiments^1–4^. Besides good veterinary practice and refinement measures, such as gentle handling, the administration of analgesic drugs during painful procedures and states plays a crucial role, particularly with surgical procedures. Apart from general anesthesia and systemic analgesia, local anesthesia, i.e. inducing the loss of nociception and therefore pain sensation in a defined local or regional area, can be a valuable tool.

Local anesthetics block sodium-specific ion channels and inhibit sodium influx. Thus, the action potential resulting from a nociceptive stimulus (e.g., skin incision) cannot be generated, and the resulting transduction of nociception in the neurons is inhibited^5^. Information on the nociceptive stimulus is not transmitted from the stimulated region to the central nervous system. As a result, the development of pain sensation in the brain is disabled. Therefore, as part of multimodal pain management, local anesthesia may reduce the amount of systematic analgesics necessary, and their administration frequency, and provide pre-emptive analgesia^6–8^.

Typical local anesthetic substances are Lidocaine, Procaine, Bupivacaine, Ropivacaine, or Benzocaine, which belong to either the amino amide or amino ester type. These drugs can be administered individually, or in combination to achieve a superior effect, and can be applied either as topical anesthesia with sprays or creams, injected in the designated area as a skin infiltration, as a nerve block, or as an epidural/spinal injection^9^. The anesthetic drug can also be administered directly on a wound via a splash dispensation or drug-soaked sponge.

In human medicine, as well as in companion or farm animal medicine, multimodal pain management comprising systemic analgesia and local anesthesia is very common for surgical procedures^10–12^. Also, “The World Small Animal Veterinary Association” recommends local anesthesia as part of multimodal pain management in small animals^13^.

Recommendations on surgical local anesthesia use in laboratory rodents are available, mainly endorsing Lidocaine and Bupivacaine. Lidocaine is a fast-acting local anesthetic with a time of onset of between 1 and 5 min and a rather short duration of action of 1–2 h, whereas Bupivacaine has a slower onset of 10–15 min but a longer duration of up to 8 h^9^. In combination, they can provide fast, long-lasting regional pain blocking. Dosage recommendations can be found in publications and university guidelines on laboratory animal pain management and vary widely from 1 to 8 mg/kg for Bupivacaine and from 3 to 10 mg/kg for Lidocaine^14–22^. The German Society of Laboratory Animal Science (GV-Solas) recommendation from 2015 does not state any dosage recommendations for local anesthesia but promotes the use of Lidocaine and Bupivacaine as part of multimodal analgesia^23^. Unfortunately, it is unclear if the above-mentioned recommendations are based on expert experience or systematic studies on efficacy and dosage finding studies in specific species as no primary studies are cited^14–23^.

Surprisingly, although local anesthetics are used extensively in larger animal species, their use in rodent surgical procedures, as well as for companion animals in veterinary practice, is reported infrequently in biomedical research^16^. Reviews of publications in different periods describing surgical procedures in rodents found a local anesthetic use of 0–10%^24–27^. Herrmann and Flecknell retrospectively reviewed 506 animal research applications sent to German authorities for approval in 2010 containing 684 surgical interventions on rats and mice. Perioperative local anesthetic use was proposed in 10–13% of these procedures^28^. In small animal veterinary practice, individual procedures like dental extractions or onychectomies are often conducted with local anesthesia; nevertheless, the overall administration of these drugs in surgery for cats and dogs is rather low, ranging from 10.5 to 55%^29–34^.

On the one hand, the lack of studies reporting the use of local anesthesia, and the lack of recommendations citing systematic studies on the effects of local anesthesia, may be due to a reporting problem with missing information in “Methods” section^26,27^. On the other hand, local anesthetics could in fact be underused and consequently represent a neglected opportunity to alleviate pain in surgical rodent models. Researchers may hesitate to use local anesthetics due to the absence of similar studies reporting their use, the lack of efficacy studies, and fear of side effects like systemic toxicity or wound healing disorders. As a result, a possible opportunity to refine surgical interventions could be missed.

In the present study, we investigated the possible benefits and adverse side effects of locally infiltrated anesthesia (Lidocaine and Bupivacaine) combined with systemic Paracetamol in mice undergoing laparotomy. We used several physiological and behavioral measurements to assess post-surgical pain and general post-surgical condition and recovery. In all animals, body weight and food intake were measured as parameters of general well-being. Water intake was assessed to calculate the intake of Paracetamol administered via drinking water. Burrowing and nest-building behavior were used to detect the reduced well-being and impairment of general condition that occurs with painful procedures^35–37^. Home cage activity was measured because activity changes can hint at impaired well-being or health state^38^. The Mouse Grimace Scale (MGS) and the von Frey test—the latter as an indicator of pain to assess hypersensitivity locally on the surgical wound area—were used to grade post-surgical pain^39,40^. Fecal corticosterone metabolites (FCMs) were analyzed to monitor adrenocortical activity and stress hormone release^41^. Finally, anhedonia, which can occur with pain or stress and is often associated with decreased intake of palatable substances, was investigated by measuring sugar consumption^42^. We hypothesized that local anesthesia in combination with Paracetamol results in reduced signs of post-surgical pain and impairment when compared to the administration of local anesthesia or systemic analgesia alone during surgery under Sevoflurane anesthesia in mice. Additionally, we hypothesized that local anesthesia does not lead to obvious adverse side effects such as disorders in wound healing.

## Methods

### Ethics statement

The Cantonal Veterinary Office, Zurich, Switzerland, approved animal housing and experimental procedures under license no. 097/2017. These were in accordance with the Swiss Animal Protection Law and conform to European Directive 2010/63/EU of the European Parliament and the Council on the Protection of Animals used for Scientific Purposes. The manuscript was prepared according to the ARRIVE guidelines^43^.

### Animals

A total of 83 female and 81 male C57BL/6J (females from surplus in-house breeding and Charles River Laboratories Sulzfeld Germany, male mice from Charles River Laboratories) were obtained. Four animals (one male and three females) died during anesthesia. A total of 80 mice of each sex were included in the analyses. Additionally, after the first evaluation of data, another control group was added (8 female and 10 male mice, Charles River Laboratories). All animals were obtained at the age of 8–10 weeks.

### Standard housing conditions

**Figure 1:**
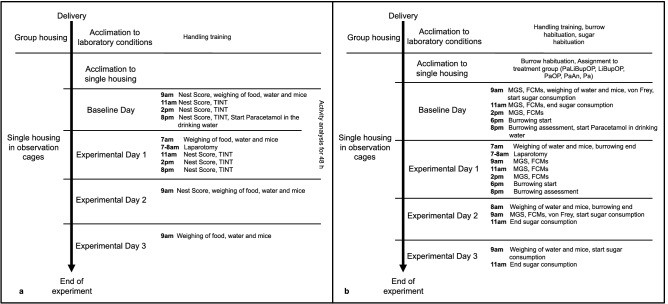
Experimental schedule displaying the procedures in both setups, such as the acclimation to laboratory conditions and single housing or handling training, as well as procedures uniquely performed in setup 1 (**a**) or setup 2 (**b**). *TINT *time to integrate nest material into the nest test, *MGS *Mouse Grimace Scale, *FCMs *fecal corticosterone metabolites. In both setups, *n* = 10 mice were assigned to PaLiBupOP, LiBupOP, PaOP, and PaAn groups. In setup 2, Pa was augmented with *n* = 8 female mice and *n* = 10 males.

Mice were acclimated in groups of four in Eurostandard Type III open-top, clear plastic cages with a wire cover (Techniplast, Hohenpeissenberg, Germany) with 21 ± 1 °C, 45 ± 5% humidity, and a light/dark cycle of 12 h/12 h (lights on at 8 a.m.) for 2 weeks after delivery (Fig. 1). The cages were filled with autoclaved dust-free sawdust bedding (80–90 g/cage; LTE E-001 Abedd, Indulab, Gams, Switzerland) and equipped with a cardboard hut (Ketchum Manufacturing, Brockville, Canada) and a red plastic house (Techniplast, Hohenpeissenberg, Germany). One tissue paper, two cotton nestlets (5 cm × 5 cm, Indulab, Gams, Switzerland), and paper fibers (Enviro-dri, Shepard Specialty Papers, Kalamazoo, MI, USA) were provided. Female mice received a wooden enrichment tool and platform (Abedd, Vienna, Austria). Food (Kliba Nafag no. 3436, Granovit AG, Kaiseraugst, Switzerland) and drinking water were provided ad libitum. From the start of acclimation, a clear handling tunnel was provided in each group cage (Datesand group, Stockport, United Kingdom). During the second week, animals were subjected to tunnel handling training with the familiar cage tunnel according to Hurst et al. for about 5 min per cage and day^44^. During subsequent experiments, animals were tunnel handled only.

A health surveillance program was performed according to FELASA guidelines. The mice were free of all pathogens listed in FELASA recommendations^45^.

### Anesthesia and analgesia treatment groups

Mice were allocated randomly to four different treatment groups (each containing 10 animals per sex and setup). Experiments were conducted in two setups. Three groups underwent anesthesia and surgery and were treated with: (i) Paracetamol, Lidocaine, and Bupivacaine (PaLiBupOP); (ii) Lidocaine and Bupivacaine (LiBupOP); or (iii) Paracetamol (PaOP). Another group underwent anesthesia only and was treated with Paracetamol (PaAn). Additionally, to test for side effects of Paracetamol, we added a group that only received Paracetamol in the drinking water (Pa) and tested this group in setup 2. These animals were added subsequently as a standalone group as the first results from setup 2 indicated an influence of Paracetamol treatment.

Paracetamol (Dafalgan, 30 mg/ml, Bristol-Myers Squibb SA, Steinhausen, Switzerland) was used as systemic analgesia in the drinking water. The dosage sought was 200 mg Paracetamol/kg body weight^14^. Assuming a water intake of at least 3 ml per 24 h in a healthy adult mouse we used 4 mg/ml Paracetamol in the drinking water. Treatment with Paracetamol started on the evening before surgery.

### Experimental schedule

Mice were separated and housed individually in observation cages (Eurostandard Type III). Animals were allowed to accustom to laboratory conditions and single housing for 24 h before baseline measurements (Fig. 1). We conducted two different experimental setups, which were performed successively, with different animals. As we applied several parameters, this separation became necessary to ensure that each single experimental parameter could be assessed properly while at the same time avoiding any implications for the animals of applying too many tests. In both experimental setups, mice and drinking bottles (for daily water intake) were weighed daily with a precision scale. The full methods for each setup are displayed in Fig. 1 and described in the following sections. Experiments were conducted by female researchers. In both setups, the cages contained the usual bedding material and one cotton nestlet. A drinking bottle with a bent sipper including a marble to reduce spillage was attached to the outside wall (300 ml bottle, TD200 nipple, UNO BV, Netherlands). Animals were tunnel handled with an individual tunnel that was not present in the cage to avoid implications with the experimental setup.

As the experiment ended, the 28 female mice from the PaAn and Pa groups were subjected to a health check and rehomed to private owners. The remaining female mice of the surgery groups were euthanized in deep Sevoflurane anesthesia by exsanguination via heart puncture. Livers were distributed within the facility via the organ sharing platform Animatch. These organs were used to establish a new method in an unrelated study to meet the reduction principle of the 3Rs. After experimental separation, male mice could not be regrouped. They were anesthetized with Sevoflurane, at least 200 µl of blood was withdrawn from the heart and cervical dislocation was conducted. The blood was used in another unrelated study not conducted by our group to characterize microRNAs in different animal models.

### Surgical procedure

Mice were weighed and, in setup 1, the hair at the surgical site was clipped (animals of setup 2 were hair clipped before for baseline von Frey test). Animals were transported in the observation cage to the operating room next door. Anesthesia was induced and maintained via nose mask [5–8% Sevoflurane (Sevorane, Abbvie, Baar, Switzerland)], 600 ml/min oxygen, flow-controlled via a Datex Ohmeda TEC 5 vaporizer, GE Healthcare, Chicago, USA]. On a warming plate (Gaymar, TP500, Orchard Park, NY, USA) set at 38 ± 1 °C, females were placed on their abdomen, males were placed on their back. The surgical site was disinfected with an alcoholic povidone-iodine solution (Braunoderm, Braun AG, Melsungen, Germany), and eyes were covered with eye ointment (Vitamin A Blache, Bausch & Lomb, Zug, Switzerland). According to the treatment group, mice were injected s.c. with a mix of Lidocaine (10 mg/kg; Kantonsapotheke Zürich, Zurich, Switzerland) and Bupivacaine (3 mg/kg; Carbostesin, AstraZeneca, Zug, Switzerland) diluted in the same amount of NaCl at six locations in a circle around the incision site. In females, the skin and muscle were incised vertically in the left flank of the mouse to a maximum length of 1 cm. The underlying fat was lifted, stretching ovary ligaments to mimic embryo transfer. In males, a 1 cm transverse incision was made through the ventral skin and abdominal muscle. The testes were exteriorized, applying a stretch on the ligaments to mimic vasectomy. The organs were relocated, and the abdominal muscle was closed with sutures (Vicryl, 6/0, Ethicon Ltd, Norderstedt, Germany). In females, skin was closed using skin staples (Precise, 3M Health Care, St Paul, MN, USA), in males using a suture (Vicryl, 6/0, Ethicon Ltd). Surgery was completed within 6–7 min, anesthesia lasted 8–9 min. Animals recovered for 10 min on the warmed surface followed by 60 min in a warming cabinet (32 °C).

### Assessment of wound healing

During daily weighing, the wound and surrounding area were checked for bleeding, discoloration, discharge, lesions, and missing sutures or staples*.*

### Setup 1

During the experiment in setup 1 (Fig. 1a), mice were housed individually in observation cages with raised plastic walls (465 mm in height) without a grid. The cages contained the usual bedding material, one cotton nestlet, and a drinking bottle attached to the outside wall. The familiar diet was provided on the cage floor.

#### Food intake

In setup 1, the daily food intake was measured by weighing the food pellets on a precision scale. The reduction of food pellets was calculated and noted as food intake in grams per 24 h. The potential loss of particulates was not accounted for.

#### Activity analysis

For 48 consecutive hours, 24 h at baseline and 24 h post-procedure, mice in setup 1 were filmed individually to assess distance moved. The cage was filmed from 60 cm above using infrared-sensitive cameras (digital video camera, Ikegami Electronics, Germany; Varifocal lenses, Computar, USA). In the dark phase, light was provided by infrared lights. An automated tracking software (Ethovision 8.5 XT, Noldus, Wageningen, The Netherlands) assessed the distance moved (center point movement) for each animal.

#### Nest building

The observation cages were equipped with a nestlet. During acclimation to single housing, mice were left undisturbed and the first assessment point for baseline nest complexity followed the next morning after 24 h. Then, mice were again provided with a new nestlet and the complexity of the built nest was assessed at several baseline time points during the day. Immediately after surgery or anesthesia, animals were provided with a new nestlet and nest complexity was assessed at the same time points during the day. Scoring was carried out as described by Jirkof et al. using a six-point scale^36^.

#### TINT

The time to integrate nest material into the nest test (TINT) was applied according to Rock et al.^37^. At several time points, animals were provided with four strips of nesting material (Enviro-dri, Shepard Specialty Papers, Kalamazoo, MI, USA). TINT was scored after 10 min as either positive (additional nesting material was integrated into the existing nest) or negative (not integrated into the existing nest). Additionally, the latency to the interaction with the material (holding with forelimbs, rolling, and intensive sniffing) was assessed from the activity analysis video material.

### Setup 2

In setup 2 (Fig. 1b), the raised plastic walls were replaced with the standard grid. The cages contained the usual bedding material, one cotton nestlet, and a drinking bottle attached to the outside wall. The familiar food was provided in the food hopper. In setup 2, the standalone Paracetamol-only group was added subsequently as the first results indicated an influence of Paracetamol treatment. In this case, full blinding for all measurements was not possible but measures were taken to ensure blinded scoring of the Mouse Grimace Score.

#### Burrowing

Burrowing behavior was tested according to Jirkof et al.^35^. Each animal was provided with a tube-like apparatus (250 ml drinking water bottle; 15 cm in length, 5.6 cm diameter on closed-end, 3 cm diameter on open-end) filled with pre-weighed food pellets (150 g ± 10 g) 2 h before the beginning of the dark phase (18:00, labeled as B 18 for baseline and PP 18 for post-procedure measurements). The tube was weighed after 2 h (20:00, labeled as B 20 for baseline and PP 20 for post-procedure measurements) and at the end of the dark phase (8:00, labeled as B 8 for baseline and PP 8 for post-procedure measurements) to assess the amount of removed food pellets and evaluate burrowing performance. Before baseline measurements, animals were habituated to the test by being presented with the filled burrowing tunnel for one night once during group-housing and once on the day mice were separated.

#### Mouse Grimace Scale

**Figure 2:**
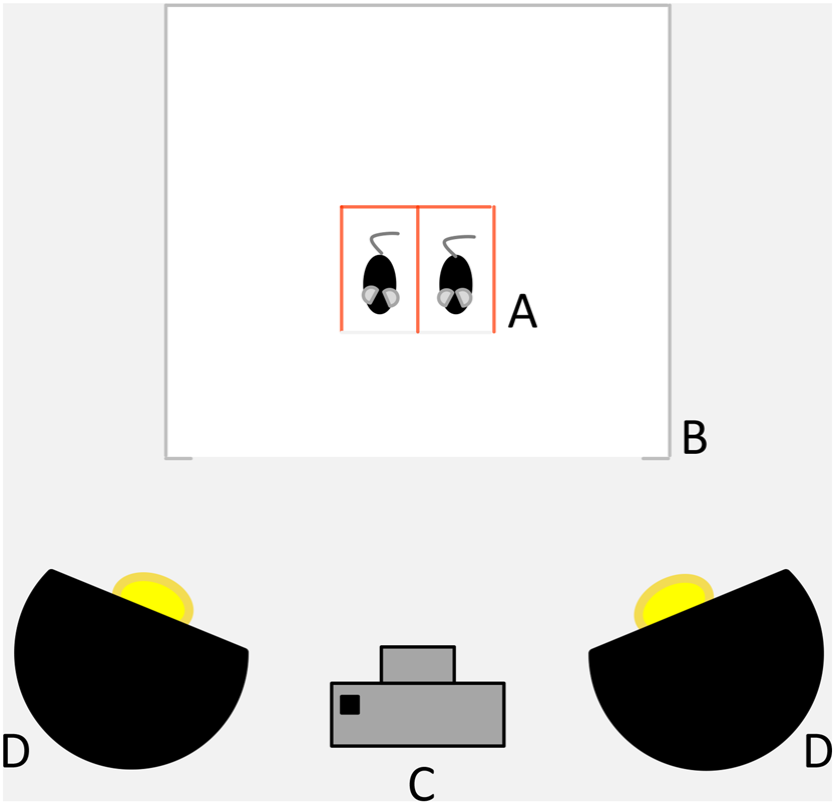
Schematic display of Mouse Grimace Scale assessment. Mice are placed in polycarbonate boxes (**A**) within a white light tent (**B**). They are filmed for 5 min by a digital camera (**C**) while being illuminated at an angle from the front by two light bulbs (**D**).

Mice were allowed to acclimate for 2 min in polycarbonate boxes (colorless-transparent front and back, red-transparent walls, 9 × 5 × 5 cm) and were then filmed for 5 min within a white light tent (80 × 80 × 80 cm) with a digital single-lens reflex camera (distance to mice 50 cm, Canon EOS 750D). Illumination was provided from an angle with two light bulbs as depicted in Fig. 2 (distance to animals 55 cm, 135 W). An automatic frame production and selection software produced 5 pictures per animal^46^, which were randomized automatically and scored by two blinded researchers according to Langford et al.^39^. Individual picture ratings where the mean MGS differed > 0.5 between raters were rare, and were discussed until agreement was reached. Images from the standalone Paracetamol group were incorporated in the analysis of previously gathered MGS images from other treatment groups to ensure blinding.

#### Von Frey

The hair around the wound sites was clipped 2 h before undertaking baseline von Frey measurement to ensure comparable conditions for baseline and post-procedure measurements. After MGS filming, females in the MGS boxes were covered with a wire grid (mesh opening 0.3 mm). Males were put on the wire grid and covered by the MGS box. Hypersensitivity of the wound site to mechanical stimuli was quantified by assessing the nociceptive behaviors^47^. After an acclimation time of 3 min, the filament (strength 4, bending force 1 g) was applied manually on the surgical site five times within 5–10 s and after a rest of 1 min another five times, for a total of 10 applications. The nociceptive behavior was scored for each application of the filament (0 = no response, 1 = immediate scratching/licking of the stimulated site, 2 = strong retraction of the abdomen or jumping). The resulting scores for the 10 challenges were added to give a total reported score.

#### Fecal corticosterone metabolites (FCMs)

From MGS boxes, feces of each animal were collected and stored at − 20 °C. The fecal samples were processed according to Touma et al.^41,48^ and concentrations of FCMs expressed in ng/0.05 g feces.

#### Sugar consumption

To assess anhedonia, sugar intake was measured. To get mice habituated to the sugar, a sugar cube (Cristal sugar cubes, Migros, Switzerland, 1.5 × 0.5 × 0.5 cm) was given to the group-housed mice three times before the experimental procedures. For testing, the mice were given a cube of sugar in the observation cage for a period of 2 h. Sugar consumed (in grams) was assessed by weighing the cube before and after the period.

### Statistical analysis

A sample size calculation was performed with G*Power^49^. The number of animals included in each test is stated either in the text or in the figure legend. Statistical analysis was carried out with R (version 3.6.1)^50^. Data were first analyzed using a linear mixed-effects regression model (*lmer*) from the *lme4* package to detect the influence of the factors treatment, time, and sex^51^. Treatment group, sex, and time of measuring (day and time point during the day) were treated as fixed effects, while the individual animal (animal ID) was treated as a random effect. Following the general analysis, the treatment and time differences were further analyzed within one sex. Differences between treatment groups were analyzed with pairwise post hoc tests using the Bonferroni correction to adjust for multiple comparisons. Differences between the baseline and the post-procedure measurements within a single treatment group were analyzed with Dunnett’s test. Degrees of freedom were approximated using the Kenward–Roger approximation. Differences were considered significant when p ≤ 0.05 (*).

## Results

In the following, we describe the influence of the factors sex, treatment, and time on each parameter. Subsequently, we compare treatment groups, as well as baseline with post-procedure measurements for each sex. Additional graphs and exact p-values for significant results from Bonferroni corrected post hoc comparison of treatment groups and Dunnett’s post hoc test of comparing baseline with post-procedure measurements can be found in the supplementary material.

### Sufficient analgesia intake was reached in most animals

Water intake was measured daily by weighing the water bottles to calculate Paracetamol intake. Absolute values can be found in Supplementary Table S1. The linear mixed-effects regression model applied detected no main effects of treatment, sex, or time. Only the combination of these factors influenced water intake significantly, which was investigated with the following post hoc tests.

Pairwise post hoc comparison of treatment groups with Bonferroni correction showed that, in male mice, the baseline intake values did not differ between treatment groups. The female animals in the Pa group had a higher baseline intake than those belonging to the LiBupOP group. In the 24 h after the procedure, male mice in the Pa group drank significantly more water than all other treatment groups. During the same period, female mice in the Pa group drank significantly more water than those in the PaLiBupOP and PaOP groups; female mice in the LiBupOP group drank more than those in the PaOP group.

Dunnett’s post hoc test revealed a significantly lower water intake 24 h post-procedure in male PaLiBupOP and PaAn and in female PaLiBupOP, PaOP and PaAn compared to baseline. At 48 h post-procedure, all groups reached baseline levels again. For details, see Supplementary Tables S2 and S3.

**Figure 3:**
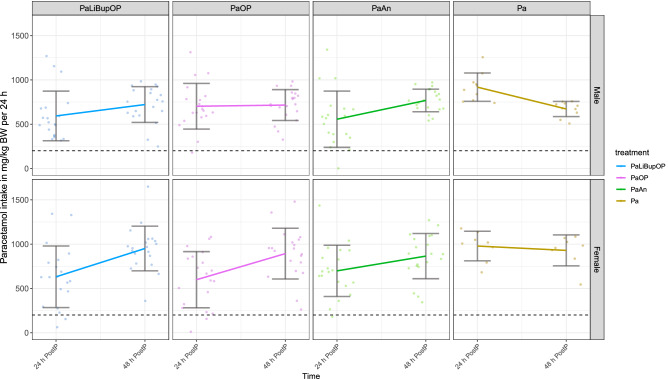
Paracetamol intake in mg/kg body weight per 24 h for male and female mice in each treatment group that received Paracetamol. Data are shown as a scatter dot plot with mean ± SD for the first 24 h (24 h post-procedure) and the second 24 h after the procedure (48 h post-procedure). The dashed line marks the target dose of 200 mg/kg body weight per 24 h. Numbers per group: PaLiBupOP, PaOP and PaAn *n* = 20/each sex; Pa male *n* = 10, Pa female *n* = 8.

Paracetamol intake was calculated by measuring the amount of medicated water consumed per day and body weight (mg/kg body weight) and is shown in Fig. 3 for animals in setup 1 and 2. The target dose of Paracetamol is 200 mg/kg body weight per 24 h, which most of the animals reached after surgery (indicated with a dashed line). The range of Paracetamol intake during 24 h was high in both male (range first 24 h: 1340.4, second 24 h: 735.2) and female (range first 24 h: 1424.6, second 24 h: 1387.1) animals.

Seven animals with either surgery or anesthesia did not reach the target dose of 200 mg/kg (indicated with the dashed line) in the first 24 h after the procedure. In the second 24 h after surgery, all animals had a Paracetamol intake of over 200 mg/kg body weight per 24 h.

### Indicators of general condition were affected post-procedure but no difference between analgesia protocols was indicated

**Figure 4:**
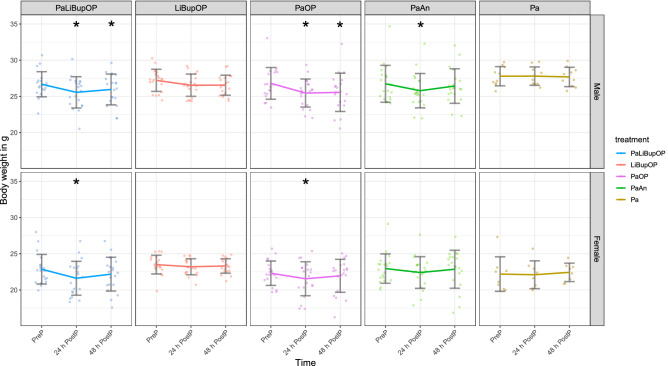
Absolute body weight in grams measured immediately before surgery/anesthesia (Pre-procedure) and 24 h and 48 h after. In both sexes, the Bonferroni post hoc test detected no differences between the treatment groups at Pre-procedure. In male mice, the Bonferroni post hoc test revealed significantly lower body weight in PaOP compared to Pa at 24 h post-procedure. Significant differences to the respective baseline measurements within one treatment group are indicated with *p ≤ 0.05, Dunnett’s post hoc. Data are shown as scatter dot plot with mean ± SD. Numbers per group: PaLiBupOP, LiBupOP, PaOP, and PaAn *n* = 20/each sex; Pa male *n* = 10, Pa female *n* = 8.

The results of daily measured body weight for setup 1 and 2 are displayed as absolute values in grams in Fig. 4. With the applied linear mixed-effects regression model we found a main effect of sex with lower body weights in females (p < 0.0001). The combination of sex, time, and treatment influenced body weight significantly, which was investigated with the following post hoc tests.

Pairwise post hoc comparison of treatment groups with Bonferroni correction showed a significantly lower body weight in PaOP males compared to Pa males 24 h post-procedure.

Dunnett’s post hoc test detected significantly lower body weight 24 and 48 h post-procedure compared to pre-procedure in male mice of the PaLiBupOP and PaOP groups. 24 h post-procedure male PaAn animals showed a significantly lower body weight than at pre-procedure. In females, PaLiBupOP and PaOP had significantly lower body weight 24 h post-procedure compared to pre-procedure. For details, see Supplementary Tables S4 and S5.

Food intake was measured by weighing food pellets every 24 h for animals in setup 1, and is displayed in Supplementary Figure S1 in grams per 24 h for each group 24 h after surgery/anesthesia (24 h post-procedure) and the second 24 h after surgery/anesthesia (48 h post-procedure).

In the linear mixed-effects regression model, a main influence of time with lower food intake post-procedure (p < 0.01) was detected. The combination of sex, time, and treatment influenced food intake significantly, which was investigated with the following post hoc tests.

Pairwise comparisons of the treatment groups with Bonferroni correction detected no significant differences in baseline food intake in both sexes. In male mice, food intake in the LiBupOP group was significantly higher than in the PaLiBupOP and PaOP groups 24 h post-procedure. The 48 h post-procedure food intake in the LiBupOP group was significantly higher than in PaOP. In female mice, significantly higher food intake was detected 24 h post-procedure in the LiBupOP group compared to the PaLiBupOP group.

Dunnett’s post hoc test of repeated measurements within each treatment group revealed a significantly lower food intake after surgery or anesthesia compared to baseline in male mice (treatment groups PaLiBupOP, PaOP, and PaAn) and in female mice (PaLiBupOP and PaOP). For details, see Supplementary Figure S1 and Tables S6 and S7.

### Mouse Grimace Scale and von Frey test indicated no differences between analgesic protocols

**Figure 5:**
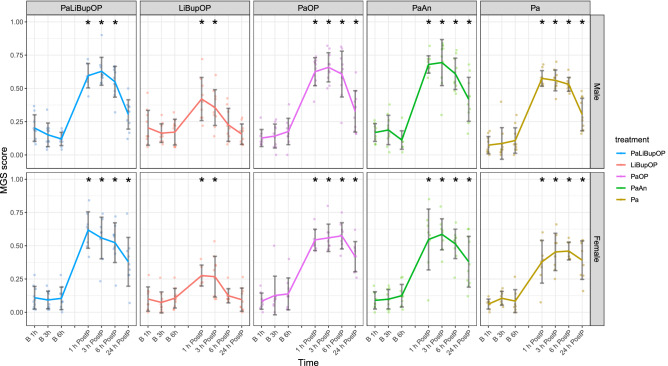
The Mouse Grimace Scale score shown for male and female animals. Values 1, 3, 6, and 24 h after the procedure and the respective baseline (B) values from the day before are displayed. The number of observations for each time point is indicated as dots. Difficulties in obtaining high-quality images for scoring resulted in lower observation numbers than actual animal numbers at several time points. Bonferroni post hoc test detected no differences between the treatment groups at baseline measurements. In male mice, LiBupOP showed significantly higher MGS compared to other treatment groups at all post-procedure time points (p < 0.05). In female animals 1 h post-procedure, MGS was significantly lower in the LiBupOP group compared to the PaLiBupOP, PaOP, and PaAn groups. Pa showed significantly lower MGS at 1 h post-procedure compared to the PaLiBupOP and PaAn groups. At 3, 6 and 24 h post-procedure LiBupOP had a significantly lower MGS than all other treatment groups. Significant differences to the respective baseline measurements are indicated with *p ≤ 0.05, Dunnett’s post hoc. Data are shown as scatter dot plot with mean ± SD. Numbers per group: PaLiBupOP, LiBupOP, PaOP, and PaAn *n* = 10/per sex; Pa male *n* = 10, Pa female *n* = 8.

The Mouse Grimace Scale was conducted to assess pain and effectivity of analgesia after laparotomy for animals in setup 2. The 1, 3, 6, and 24 h post-procedure MGS scores as well as the respective baseline values are displayed in Fig. 5. With the linear mixed-effects regression model, a significant influence of time on the MGS was detected with significantly higher values post-procedure (p < 0.0001). The combination of sex, time, and treatment influenced the MGS significantly, which was investigated with the following post hoc tests.

Pairwise comparisons with Bonferroni correction showed that the baseline MGS did not differ between treatment groups. In male animals, a significantly lower MGS score was detected in LiBupOP compared to the other treatment groups at all post-procedure time points. In female animals 1 h post-procedure, MGS was significantly lower in the LiBupOP group compared to PaLiBupOP, PaOP, and PaAn groups and significantly lower in Pa compared to PaLiBupOP and PaAn groups. At 3, 6, and 24 h post-procedure, LiBupOP had a significantly lower MGS than the other treatment groups.

In male animals, post hoc testing with Dunnett’s correction detected significantly higher MGS 1 and 3 h post-procedure compared to the respective baseline values in all treatment groups. At 6 h post-procedure only LiBupOP, and 24 h post-procedure only PaLiBupOP and LiBupOP, were back at baseline values. In female animals, all treatment groups had significantly higher MGS at 1 and 3 h post-procedure compared to baseline, whereas at 6 and 24 h post-procedure only LiBupOP was back at baseline values. For details, see Supplementary Tables S8 and S9.

The von Frey test was conducted to assess hypersensitivity around the surgical wound area for animals in setup 2. Results for baseline and post-procedure measurements are depicted in Supplementary Figure S2. In general, there was no influence of treatment, sex, time, or their combination on the von Frey score.

Pairwise comparisons of treatment groups with Bonferroni correction showed no significant differences in the baseline von Frey score. In the male Pa group, the post-procedure von Frey score was significantly lower compared to the PaLiBupOP and PaOP groups. Post hoc comparison with Dunnett’s correction detected significantly higher von Frey score post-procedure compared to baseline in male animals of the PaLiBupOP and PaOP groups. For details, see Supplementary Figure S2, Tables S10 and S11.

### Distance moved was reduced post-procedure in all groups but no difference between analgesic protocols was indicated

The distance moved of animals in setup 1 was measured during 24 h before and after surgery or anesthesia to assess activity (Supplementary Figure S3). We detected an influence of time (p = 0.02) with lower values 24 h post-procedure on the distance moved with the linear mixed-effects regression model.

In both sexes, Bonferroni post hoc comparisons detected no differences between treatment groups at baseline level. Within males, the LiBupOP group had a significantly higher moved distance in the 24 h post-procedure compared to the PaOP group.

Post hoc comparison with Dunnett’s correction detected that, in both sexes and all treatment groups, animals had moved a significantly lower distance 24 h post-procedure when compared to baseline values. For details, see Supplementary Figure S3, Tables S12 and S13.

**Figure 6:**
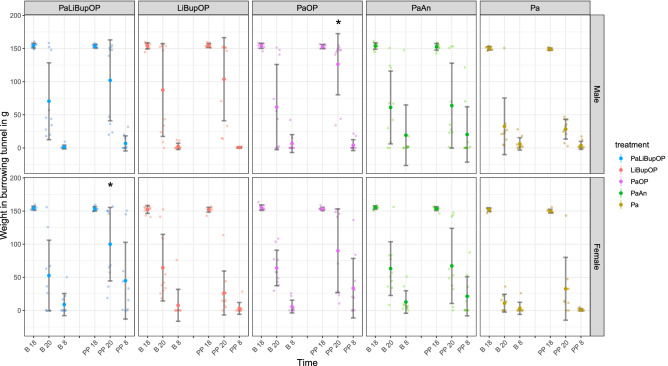
The burrowing behavior as the weight of the burrowing tunnel in grams for baseline (B) and post-procedure. Weight is shown at 2 h (B 20 and PP 20) and 12 h (B 9 and 24 h PP 8) after the start of the test (B 18 and PP 18) in male and female mice and displayed for each treatment group. Post hoc testing with Bonferroni correction was used to compare burrowing behavior between the treatment groups. Male Pa mice removed significantly more pellets from the burrowing tunnel at B20 compared to LiBupOP mice. At P20, male Pa mice removed significantly more pellets than PaLiBupOP, LiBupOP, and PaOP mice. Male PaAn animals removed significantly more pellets than PaOP animals at P20. In female animals, the Pa group removed significantly more pellets than the LiBupOP, PaOP, and PaAn groups at B20. At P20, female PaLiBupOP mice removed significantly fewer pellets than LiBupOP and Pa mice; LiBupOP mice removed significantly more than PaOP and PaAn mice; Pa mice removed significantly more pellets than PaOP. At 24 h after surgery or anesthesia (PP 8), the PaLiBupOP group removed significantly fewer pellets than the LiBupOP and Pa groups. Significant differences to the respective baseline measurements are indicated with *p ≤ 0.05, Dunnett’s post hoc. Data are shown as scatter dot plot with mean ± SD. Numbers per group: PaLiBupOP, LiBupOP, PaOP and PaAn *n* = 10/per sex; Pa male *n* = 10, Pa female *n* = 8.

Burrowing behavior was unaffected by treatment and indicated no differences between analgesic protocols Burrowing behavior was tested to detect impairments in the animal’s well-being in setup 2. The weight of pellets in the burrowing tunnel is displayed for baseline as well as post-procedure (Fig. 6). At 2 h after the test started (B 20 and PP 20), burrowing performance was variable within treatment groups, while at the endpoint 12 h after the start (B 8 and PP 8) the overall performance had improved.

For burrowing behavior, a significant influence of the combination of the factors sex, time, and treatment was detected, which was investigated with the following post hoc tests.

Post hoc treatment group comparison with Bonferroni correction showed that the male Pa group removed significantly more pellets from the burrowing tunnel at B 20 compared to the LiBupOP group. At PP 20, male Pa animals removed significantly more pellets than PaLiBupOP, LiBupOP, and PaOP animals. The male PaAn group removed significantly more pellets than the PaOP group at PP 20. In female animals, the Pa group removed significantly more pellets than the LiBupOP, PaOP, and PaAn groups at B20. At PP 20, female LiBupOP animals removed significantly more pellets than PaLiBupOP, PaOP and PaAn animals; the Pa group removed significantly more pellets than the PaLiBupOP and PaOP groups. At 24 h after surgery or anesthesia (PP 8), the PaLiBupOP group removed significantly fewer pellets than LiBupOP and Pa mice.

Post hoc comparison with Dunnett’s correction showed that male PaOP as well as female PaLiBupOP mice removed significantly fewer pellets at PP 20 than at B 20. For details, see Supplementary Tables S14 and S15.

### Nest building behavior recovered fastest in animals treated with local anesthesia after surgery

**Figure 7:**
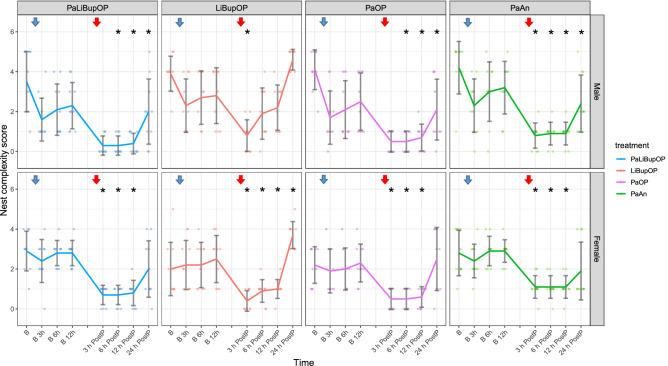
Nest complexity scores displayed for female and male mice for each treatment group. The blue arrow indicates that a new nestlet was provided for baseline measurements, the red arrow indicates the surgery/anesthesia followed by the distribution of a new nestlet. Bonferroni post hoc testing detected no significant differences between the treatment groups at baseline level and 3 h post-procedure. Male LiBupOP had a significantly higher nest complexity score than PaLiBupOP and PaOP 6 and 12 h post-procedure; at 24 h post-procedure male LiBupOP had a significantly higher score than all other treatment groups. In female mice, 24 h post-procedure nest complexity in LiBupOP was significantly higher than in all other treatment groups. Significant differences to respective baseline measurements are indicated with *p ≤ 0.05, Dunnett’s post hoc. Data are shown as scatter dot plot with mean ± SD. Numbers per group: PaLiBupOP, LiBupOP, PaOP, and PaAn *n* = 10/per sex.

Nest building behavior was assessed in each mouse in setup 1 to check for behavioral changes that could indicate decreased well-being. The results of baseline and post-procedure measurements (Fig. 7) show the variability of scores and their time course. The baseline measurement (B in Fig. 7) shows the nest complexity score in the morning when mice were undisturbed for 24 h to build a nest. Immediately after this scoring, nesting material was removed and a new nestlet was provided (see blue arrow, Fig. 7) to score baseline values of the newly built nest. Animals also received a new nestlet immediately after surgery or anesthesia (see red arrow, Fig. 7).

In the nest complexity score, the linear mixed-effects regression revealed an influence of time with significantly lower values after the procedure (p < 0.0001). The combination of sex, time, and treatment influenced the nest complexity significantly, which was investigated with the following post hoc tests.

No significant difference between treatment groups at baseline time points was detected with post hoc comparison with Bonferroni correction. Male LiBupOP animals had a significantly higher nest complexity score than their PaLiBupOP and PaOP counterparts at 6 and 12 h post-procedure. At 24 h post-procedure, LiBupOP mice of both sexes had a significantly higher score than all other treatment groups.

Post hoc comparison with Dunnett’s correction revealed that male LiBupOP and PaAn animals had significantly lower nest complexity scores 3 h post-procedure than at B 3 h. At 6, 12, and 24 h post-procedure, only the male LiBupOP group went back to baseline scores. In female mice at 3, 6, and 12 h post-procedure, all treatment groups had significantly lower scores than the respective baseline scores. At 24 h post-procedure, female LiBupOP mice had a significantly higher score than at the respective baseline. For details, see Supplementary Tables S16 and S17.

TINT was applied in all animals of setup 1 to detect behavioral changes that could hint at reduced well-being. Cage-side assessment of TINT after 10 min resulted in 2.5% (3 out of 120) positive baseline observations in females and 10.8% (13 out of 120) positive baseline observations in male mice overall treatment groups. In postsurgical measurements, females had a positive rate of 0% (0 out of 120), males of 1.7% (2 out of 120). Technical problems with the video recording resulted in a loss of several observations. Therefore, latency to interaction was not analyzed.

### Stress response was similar post-procedure and indicated no differences between analgesic protocols

**Figure 8:**
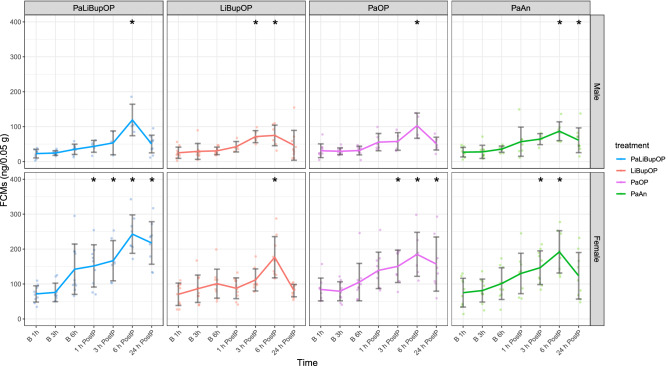
Fecal corticosterone metabolites (ng/0.05 g feces) in baseline and post-procedure phase for male and female mice in each treatment group. FCMs were measured at the respective baseline time points and 1, 3, 6, and 24 h after surgery or anesthesia. The number of animals from which samples could be collected is indicated as dots. This number is lower than the actual animal number in several cases as feces could not be obtained from all animals. Bonferroni post hoc analysis detected no significant differences between the treatment groups at the baseline level. Male PaLiBupOP had a significantly higher FCM concentration than LiBupOP 6 h after surgery. In female animals, PaLiBupOP showed significantly higher FCM levels than LiBupOP 1, 6, and 24 h after surgery. At 24 h after surgery or anesthesia, female PaLiBupOP had significantly higher FCM levels than PaOP and PaAn, while FCM concentrations in LiBupOP were significantly lower than in PaOP. Significant differences to the respective baseline measurements are indicated with *p ≤ 0.05. Data are shown as scatter dot plot with mean ± SD. Numbers per group: PaLiBupOP, LiBupOP, PaOP, and PaAn *n* = 10/per sex.

FCMs were measured in samples on baseline and post-procedure time points to assess the stress response to the procedure in animals exposed to the second setup (Fig. 8).

A general influence of sex, with significantly higher FCMs in female mice (p < 0.05), and of time, with significantly higher values after the procedure (p < 0.05), on FCMs was detected. The combination of sex, time, and treatment influenced FCMs significantly, which was investigated with the following post hoc tests.

Pairwise comparisons with Bonferroni correction revealed no significant differences between the treatment groups at baseline. Male PaLiBupOP mice had significantly higher FCM levels than LiBupOP mice 6 h after surgery. In female animals, the PaLiBupOP group showed significantly higher FCMs than the LiBupOP group at 1, 6, and 24 h after surgery. At 24 h after surgery or anesthesia, the female PaLiBupOP group had significantly higher FCMs than the PaOP and PaAn groups, while the LiBupOP group had significantly lower FCMs than the PaOP group.

Post hoc comparisons with Dunnett’s correction revealed significantly higher FCMs in male PaLiBupOP animals 6 h post-procedure compared to the respective baseline measurement. Male LiBupOP mice showed significantly higher FCMs 3 and 6 h post-procedure. The male PaOP group had significantly higher FCMs 6 h post-procedure. The PaAn group had significantly higher values 6 and 24 h after anesthesia when compared to baseline. Female PaLiBupOP animals showed significantly higher FCMs at all post-procedure time points compared to the respective baselines. Female LiBupOP mice had significantly higher FCMs compared to baseline 6 h after surgery. Post-procedure values were significantly higher at 3, 6, and 24 h post-procedure in the female PaOP group. In female PaAn animals, significantly higher FCMs compared to baseline were detected 3 and 6 h after anesthesia. For details, see Supplementary Tables S18 and S19.

### Sugar consumption was not influenced by surgery, anesthesia or analgesia

Sugar consumption was measured before, and on the 2 days following, surgery/anesthesia to test for anhedonia in the animals of setup 2. Overall, the absolute sugar intake in male and female mice changed only slightly after surgery or anesthesia but a significant influence of treatment was detected with the linear mixed-effects regression model. We saw higher values in PaLiBupOP, LiBupOP, PaOP, and PaAn groups compared to the Pa group (p < 0.05). The combination of sex, time, and treatment influenced the sugar intake significantly, which was investigated with the following post hoc tests.

Post hoc comparisons of treatment groups with Bonferroni correction revealed that male Pa mice had significantly lower sugar intake than PaLiBupOP and LiBupOP mice at baseline level and 48 h post-procedure. Post hoc comparisons with Dunnett’s correction revealed that male PaOP had a significantly lower sugar intake at 24 h post-procedure compared to baseline. For details, see Supplementary Figure S4, Tables S20 and S21.

### Wound healing was not affected by local anesthesia

Wound condition was checked daily during the weighing of the animal and after euthanasia. From a total of 120 operated mice, 7 animals (5.8%) showed irregularities. At euthanasia, one male animal of the LiBupOP group was noted to have removed its stitches. The wound was slightly encrusted but showed no signs of inflammation. On the day of surgery, two female mice of the PaLiBupOP group removed both staples. In the LiBupOP group, as well as in the PaOP group, one animal removed both and one animal removed one staple. In conclusion, from 80 animals (6.3%) that were injected with local anesthetics, 5 removed their staples or stitches but had no signs of inflammation or wound healing disorders.

## Discussion

In this study, we evaluated the possible benefits and side effects of a mixture of Lidocaine and Bupivacaine as locally infiltrated analgesia combined with systemic Paracetamol administered via drinking water after laparotomy in mice. Systemic Paracetamol, Sevoflurane anesthesia, and surgery affected corticosterone metabolites and behavioral measurements distinctly. Nevertheless, we did not observe meaningful differences in pain relief between systemic analgesia only versus systemic analgesia with local anesthesia. Mice treated with a combination of local anesthesia and systemic analgesia did not show less signs of post-surgical pain or improved post-surgical condition and recovery. All applied pain relief protocols appeared to be effective as changes after surgery were comparable to control groups. No adverse side effects of Lidocaine and Bupivacaine were observed. We detected no relevant sex differences in most of the assessed parameters.

We chose voluntary intake of analgesia to reduce stress due to animal handling. Because voluntary intake via drinking water has been criticized as insecure^52^, we measured the actual water intake carefully. We found that, in the 24 h after surgery or anesthesia, only 7 animals had a calculated Paracetamol intake of less than 200 mg/kg, while all others reached this target dose per 24 h. Overall, Paracetamol intake was variable, and several animals had a 6–7 times higher 24 h intake. Paracetamol in high dosages (250–500 mg/kg i.p. or i.v.) can be hepatotoxic and even lethal in mice^53,54^. We suspect that the unlimited access to food and the constant administration of Paracetamol via the drinking water prevented obvious Paracetamol toxicity in our study. Despite individual variability in water intake, we measured an average baseline and post-procedure water intake comparable to reported values for C57BL/6 mice^55^. We therefore assume that spillage was not a major issue in our study and that some animals indeed had a high water, and therefore Paracetamol, intake^55^. This variability of (medicated) water intake has been reported and can be considered a disadvantage of administering analgesia in the drinking water^56,57^. In our study, the Paracetamol intake was not confirmed by analysis of blood concentration. Nevertheless, given the overall high calculated Paracetamol intake exceeding the target dose of 200 mg/kg, we assume that most animals received a sufficient Paracetamol dose. This is supported by the results of the MGS, von Frey test, and nest complexity scoring, which detected no differences between the operated, anesthetized or Paracetamol-only treated animals. In conclusion, we suggest that Paracetamol was efficient in reducing pain.

To measure exact food intake, animals have to be kept in metabolic cages on a grid without bedding, which inflicts an additional burden on the mice^58^. Therefore, we chose the commonly used method of simply weighing food pellets to avoid this burden^59,60^. The calculated baseline values in our study were in the range of physiological food intake per day for adult C57BL/6 mice^55^; post-procedure intake was similar in operated animals with local anesthesia only, but decreased in animals treated with Paracetamol. We observed an unexpected high weight loss and high variability of body weight in Paracetamol-treated animals after surgery or anesthesia. This might be explained by the reduced food intake after surgery or anesthesia in these groups receiving Paracetamol compared to the group receiving only local anesthesia. Compared to studies using the same laparotomy model with Buprenorphine treatment, or conducting Isoflurane anesthesia only in C57BL/6 mice, the body weight loss in our study is rather high^56,61,62^. Other than the body weight decrease, animals showed no signs of impaired health and did not reach any humane endpoint (20% weight loss, apathy, self-mutilation, or signs of remaining pain for 3 days). Animals treated with Lidocaine and Bupivacaine only before surgery, or animals receiving only Paracetamol in drinking water without surgery or anesthesia, had a more constant body weight. In conclusion, it seems that Paracetamol treatment in combination with Sevoflurane anesthesia leads to reduced food intake and increased body weight loss.

To analyze if additional local anesthesia leads to better pain relief, all animals were tested with a range of established pain detection methods for mice. To limit confounding effects due to additional stress from various testing parameters, we conducted the different tests in two setups. In both setups, animals were weighed, and the water intake was assessed. In setup 1, we additionally assessed food intake and applied three behavioral parameters displayed in the home cage (activity, nest complexity and TINT). Animal handling was not required for these tests. In setup 2, the assessment of MGS and von Frey needed handling while the remaining two tests were done in the home cage without handling (burrowing behavior and sugar intake). Fecal samples were collected from the MGS boxes without the need for additional handling.

Mouse Grimace Scores were increased for 24 h in all groups receiving Paracetamol regardless of the subsequent intervention. In contrast, animals receiving local anesthesia as sole analgesic before surgery had the lowest increase of MGS, and MGS was increased at fewer time points. These results suggest that anesthesia, as well as Paracetamol, affect the MGS. While an influence of inhalation anesthesia on MGS is known^59,62–64^, an increase in MGS due to Paracetamol has not been reported so far. In general, there is little evidence of analgesic drugs like Tramadol or Buprenorphine affecting MGS^64–66^. In a study by Jirkof et al., mice that received a mixture of Tramadol and Paracetamol (T:P) in the drinking water with or without general anesthesia showed increased MGS^67^. In another study, the administration of Tramadol only in the drinking water did not increase MGS^59^, suggesting that Paracetamol was the substance affecting MGS. In a study by Matsumiya et al., Paracetamol injection led to the highest MGS compared to other analgesics or saline injection^63^. In contrast, Almeida et al. detected no change in MGS in control mice after intragastric gavage of a low dose of 30 mg/kg Paracetamol^14,23,68^. Taking this evidence together, Paracetamol treatment seemed to be responsible for the increased MGS observed in our study. We are not aware of a potential mechanism that could explain the observed effects of Paracetamol treatment, and this should be the subject of further investigation.

Operated and non-operated animals with Paracetamol had a comparable increase of MGS, and presurgical local anesthesia led to only a low increase of MGS in operated animals. Therefore, we suggest that all pain management protocols (systemic Paracetamol, local anesthesia, or the combination) were efficient in alleviating pain in our laparotomy model.

After a surgical incision, hypersensitivity, e.g. hyperalgesia or allodynia, around the wound area can occur. Therefore, we expected lower von Frey scores in animals that received local anesthesia before their surgery compared to the operated animals with only general analgesia^40^. Female operated animals showed no significant increase post-procedure in all groups, suggesting the successful prevention of hypersensitivity with all treatments. Nevertheless, due to the lack of an operated control group without analgesic treatment, we cannot rule out that the laparotomy failed to increase hypersensitivity in female mice in the first place. On the other hand, male mice indeed seemed to show hypersensitivity that could not be prevented by systemic analgesia. Male operated animals with local anesthesia only showed no hypersensitivity, whereas treatment with Paracetamol only, or in combination with local anesthesia, was not able to prevent the hypersensitivity. In the present study, a rather basic approach to investigate local hypersensitivity was chosen, namely an analogous von Frey device and the reaction to stimulation by one filament. A more sophisticated setup (electronic von Frey anesthesiometer) may have been necessary to detect subtle changes in hypersensitivity occurring with the rather mild laparotomy that we used. Additionally, it should be noted that assessment of the von Frey score could not be blinded in the subsequently added Paracetamol-only group and we therefore cannot rule out bias for this specific treatment group. Taken together, the gathered von Frey results were not conclusive in our study.

Locomotory activity (distance moved) was decreased in all treatment groups in the first 24 h after surgery or anesthesia—a result also frequently reported by other studies^61,66,69^. The lack of difference between control and operated groups again hints towards sufficient pain relief by all analgesia protocols.

The burrowing test results of the compared groups differed mainly at the time point 12 h after surgery or anesthesia. Overall, animals with Paracetamol only or animals undergoing surgery with local anesthesia only performed better than animals from the two groups receiving Paracetamol after surgery. The burrowing performance of the latter animals was comparable to those in the control group with Paracetamol and anesthesia, again hinting towards a sufficient pain relief after surgery. Paracetamol only in the drinking water did not affect burrowing performance, as was also reported for Tramadol but not for Buprenorphine^61,65,66^.

The nest complexity score was reduced after surgery and anesthesia in all groups—a finding often described in the literature^36,59,70^. In both sexes, animals treated with local anesthesia only recovered fastest. An alternative measure to assess nest-building activity, TINT was low in all animals at baseline as well as at post-procedure time points. This is a finding we saw in a previous study in female B6 mice^59^. Rock et al. and Häger et al. reported higher numbers of successfully integrated nesting material but, in both studies, performance might be better due to group housing^37,71^. TINT performance could also be increased by several days of training before baseline measurement^72^. Due to the low-performance level in our study, we cannot draw conclusions from our TINT results. In conclusion, the lack of difference between groups with surgery or anesthesia only in the nest complexity score indicates reliable pain relief with all treatment protocols.

To further investigate the impact of surgery and the different analgesics, we applied measurements of stress and anhedonia. Voluntary voided fecal samples were collected for corticosterone metabolite measurements while animals were observed in MGS boxes. This method was chosen to avoid additional stress due to handling and manipulation. This circumstance resulted in a low number of samples in some groups at several time points. Therefore, FCM results must be evaluated with caution. Overall, male mice showed lower concentrations and smaller changes after procedures compared to baseline measurements than females. A sex difference in FCM concentrations has been described previously by others^41,48,73,74^. We expected the peak of FCMs to occur 6–10 h after the stressful event^41,48^, namely the surgery or anesthesia. Indeed, we saw the highest FCM values in all groups at 6 h after the procedure, whereas levels decreased again after 24 h. Operated males and females treated with the combination of Paracetamol and local anesthesia had higher FCMs at several time points compared to animals with local anesthesia only. But, overall, FCMs in operated animals did not differ from animals with anesthesia only. In contrast to our results, Hohlbaum et al. reported no effect of anesthesia on FCM levels in both sexes^62^. When compared to FCM levels reported by Pfeiffenberger et al., where female mice were subjected to intra-bone marrow transplantation, the increase of FCMs in our study was rather low^75^, a plausible finding as transplantation surgery is rather invasive and long compared to our procedure. Taken together, we detected no significant differences in FCM values between the treatment groups with surgery or with anesthesia only, suggesting that the different pain management protocols relieved pain and the resulting stress. Anhedonia is associated with a reduced ability to experience pleasure. We hypothesized that unrelieved pain due to surgery leads to discomfort in mice, resulting in anhedonia that can be detected with a decreased intake of sugar. In our experiment, the intake of sugar was stable in the days after surgery or anesthesia when compared to baseline. Therefore, we suspect that neither the surgical procedure nor the anesthesia itself led to anhedonia. These results are in line with other studies on low impact procedures, e.g. sugar intake is not influenced by ear notching or different blood withdrawal techniques^42,70^. Anhedonia is more likely induced in models that impact animals for a longer time than the short-lasting discomfort and pain by surgery/anesthesia expected in our study^76,77^. In conclusion, a short-lasting procedure with low-to-moderate pain such as the laparotomy used here might not lead to anhedonia in mice.

Concerns against the use of local anesthesia are expected wound healing disorders and systemic toxicity^78^. Several studies have found histological changes in wound healing after the administration of local anesthesia in different species^79,80^, which did not necessarily result in altered wound healing^80^. Other studies found neither changes in histology nor wound healing disorders^81,82^. Due to the conflicting reports on histology and general wound healing, and because we focused mainly on the analgesic efficacy of local anesthesia, we decided on gross examination of wound healing only. In the present study, 6.3% of animals showed irregularities at the wound site, but, overall, wound healing was unsuspicious and as expected for this model. Additionally, no systemic toxic effects were observed.

To reduce overall animal numbers, we chose to use surplus animals from in-house breeding whenever possible in addition to mice from a commercial breeder. Laboratory rodent breeding currently produces high numbers of surplus animals^83^. Besides the waste of resources, this poses a great ethical problem for the scientific community. These animals were distributed equally among all groups. Using mice from different providers may bear the risk of introducing additional variables into the study, potentially resulting in increased data variability. Indeed, we observed high variability in some parameters (e.g. body weight, water intake, or burrowing behavior). However, this was detected in females as well as male mice and the latter were obtained from one commercial breeder only. Therefore, we think that the variability did not result from the fact that our female mice were from in-house breeding as well as from a commercial breeder.

The surgeries applied differ technically in female and male mice, which could raise questions about their comparability. Nevertheless, as both surgeries include skin and muscle incision, a light pull on both ovary and testicle to build tension and to create a painful stimulus, we assume that pain quality and intensity were comparable in the applied surgeries. In fact, both surgeries are graded as mildly to moderately painful and have been compared also in other studies^35,67,84^.

## Conclusion

As studies evaluating the beneficial effects of local anesthesia for mouse surgery in the context of animal welfare are not available to our knowledge, it was unclear which parameters might be suitable to measure the effects of local anesthesia. We therefore used a rather explorative approach combining several post-operative pain parameters, a hypersensitivity test, and general condition parameters in two setups. Our results indicate that variation is a challenging factor in model predictions, and that the results of statistical analyses should be interpreted carefully. Nevertheless, to our knowledge, we provide the first systematic and intensive analysis of the benefits of local anesthesia in mice, which may serve as a starting point for further studies.

In the present study, we could not demonstrate that systemic analgesia in combination with local anesthesia is superior to systemic analgesia only. On the other hand, we observed no negative side effects of added local anesthesia. Theory and published evidence from humans and other species hint at benefits of local anesthesia^6^. We cannot rule out that we may have not detected the benefits due to the unexpected side effects of Paracetamol or because our measurement parameters were not sensitive enough. For surgeries such as the laparotomy used here, our data suggest that all three protocols are effective in alleviating pain. Therefore, we should give local anesthesia the benefit of the doubt and start to integrate it into multimodal pain management protocols. Additionally, because the sham surgery conducted in this study has a very low impact, we caution to conclude that Sevoflurane and local anesthesia without systemic analgesia might be sufficient for all minor surgeries.

## Supplementary Information

Supplementary Figure S1.

Supplementary Figure S2.

Supplementary Figure S3.

Supplementary Figure S4.

Supplementary Information.

## Data Availability

The authors declare that all data supporting the findings of this study are available within the paper and its supplementary information file. Further information is made available by the authors upon request.
