# Supplementary figures and images for: Lidocaine and bupivacaine as part of multimodal pain management in a C57BL/6J laparotomy mouse model
Source: Sci Rep. 2021 May 25;11:10918. doi: 10.1038/s41598-021-90331-2 (PMC8149411; doi:10.1038/s41598-021-90331-2)

Food intake in g/24 h

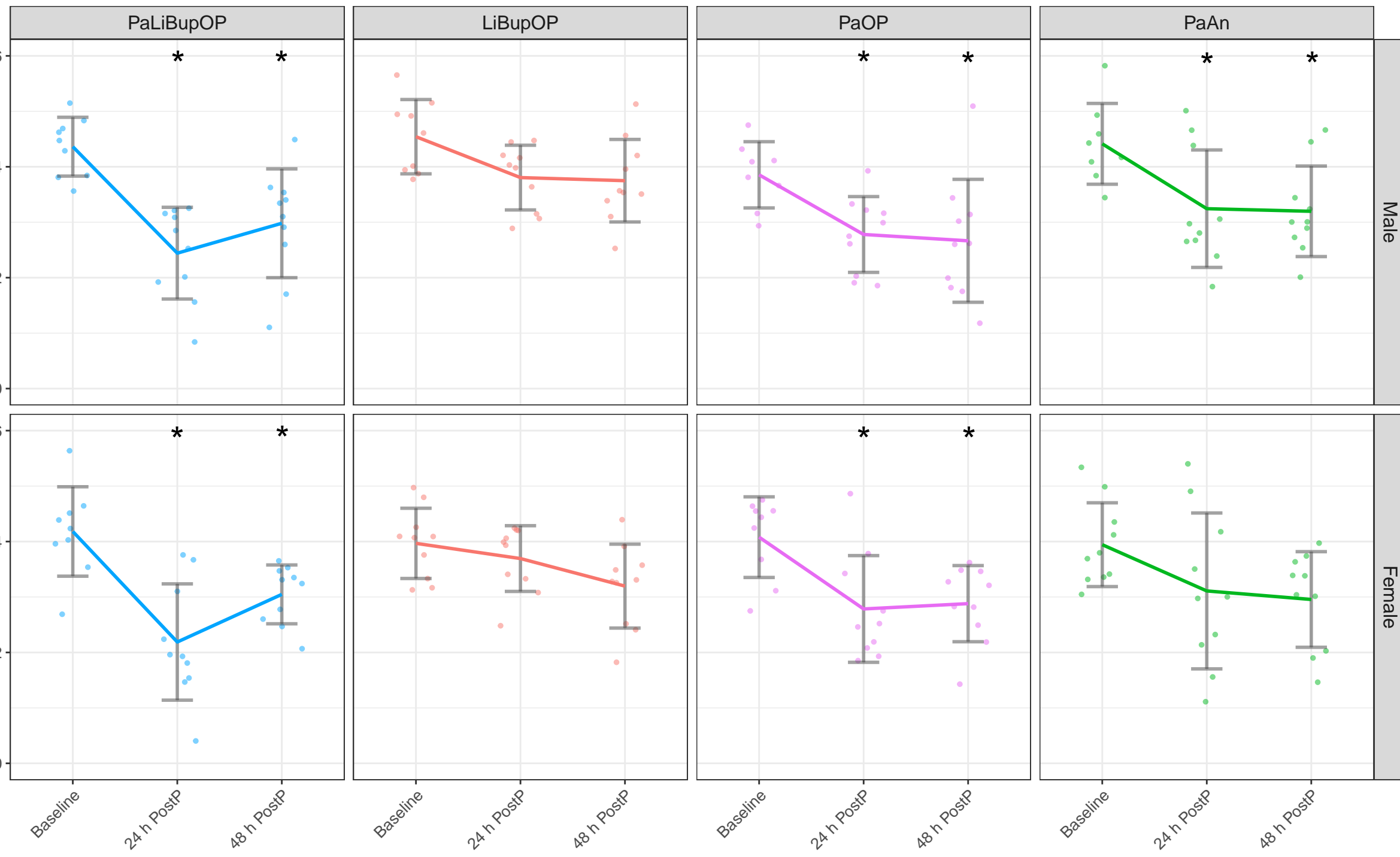

treatment

- PaLiBupOP
- LiBupOP
- PaOP
- PaAn

Time

Supplement: Supplementary file 1 — Supplementary Figure S1. [file 41598_2021_90331_MOESM1_ESM.pdf]

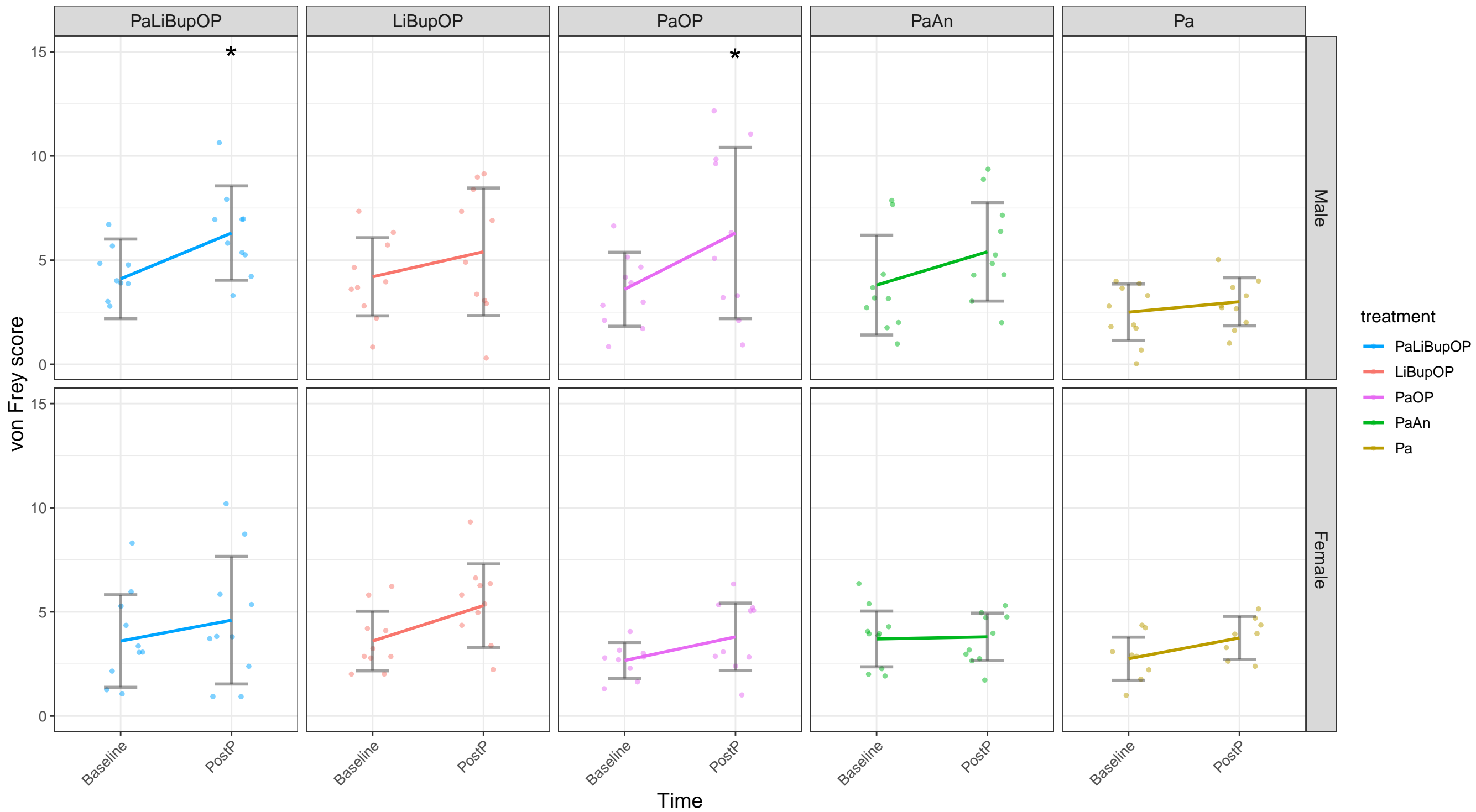

Supplement: Supplementary file 2 — Supplementary Figure S2. [file 41598_2021_90331_MOESM2_ESM.pdf]

Sugar intake in g/24 h

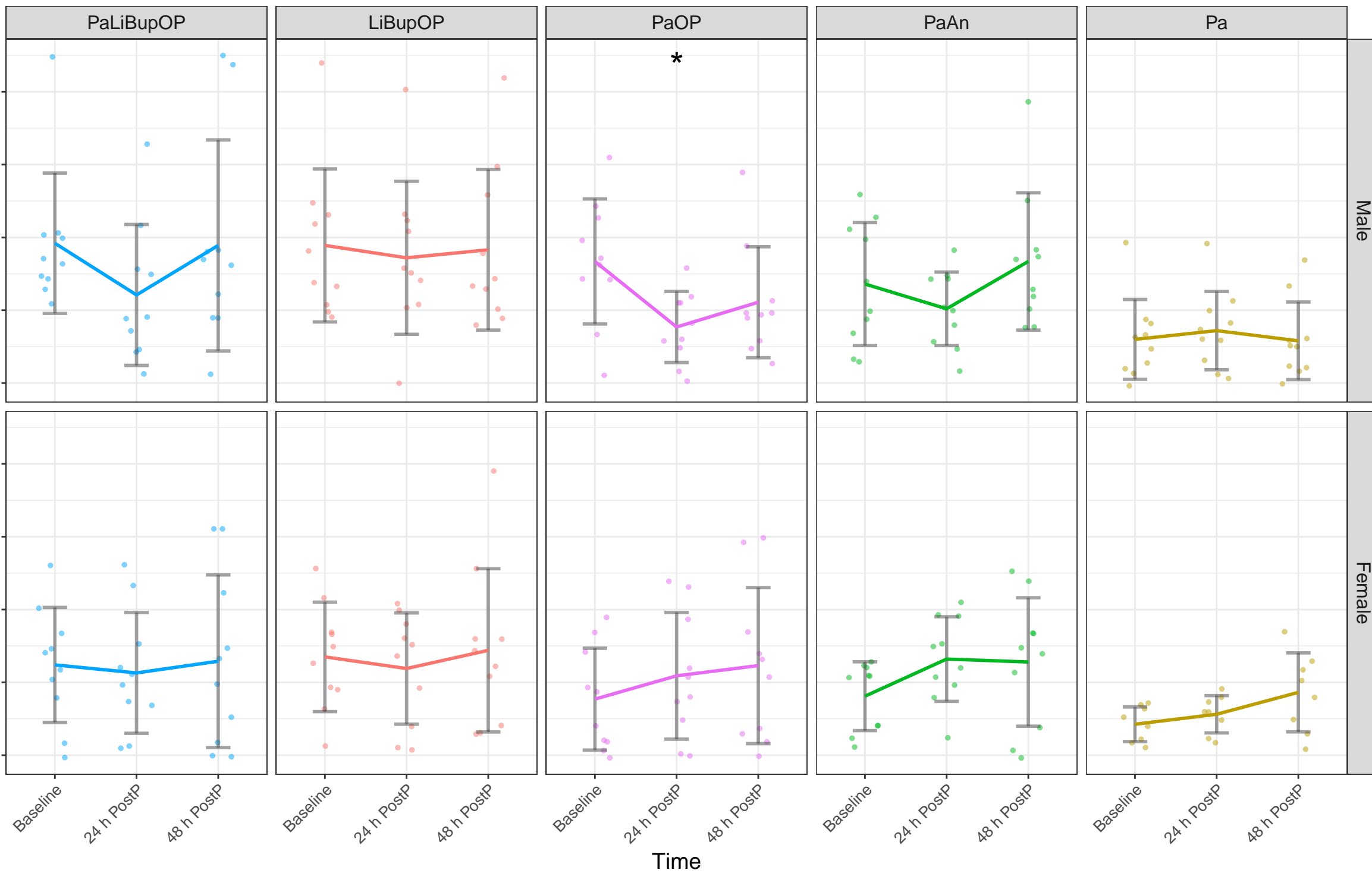

Supplement: Supplementary file 4 — Supplementary Figure S4. [file 41598_2021_90331_MOESM4_ESM.pdf]
