# Supplementary material for: Lidocaine and bupivacaine as part of multimodal pain management in a C57BL/6J laparotomy mouse model
Source: Sci Rep. 2021 May 25;11:10918. doi: 10.1038/s41598-021-90331-2 (PMC8149411; doi:10.1038/s41598-021-90331-2)
Supplement: Supplementary file 3 — Supplementary Figure S3. [file 41598_2021_90331_MOESM3_ESM.pdf]

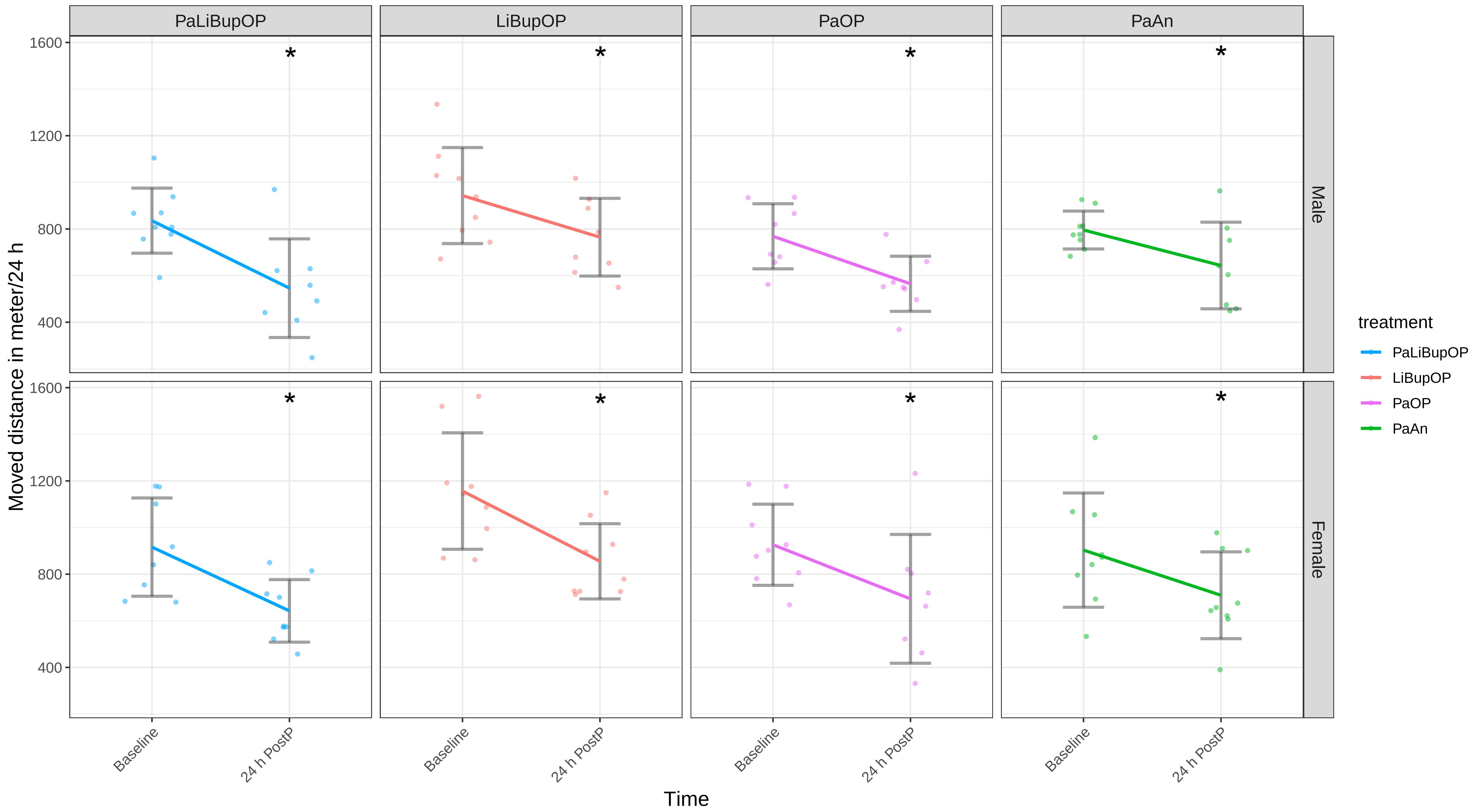

PaLiBupOP
LiBupOP
PaOP
PaAn
Male
Female
1600
1200
800
400
*
Moved distance in meter/24 h
Baseline
24 h PostP
Time
treatment
PaLiBupOP
LiBupOP
PaOP
PaAn
