# Supplementary material for: Lidocaine and bupivacaine as part of multimodal pain management in a C57BL/6J laparotomy mouse model
Source: Sci Rep. 2021 May 25;11:10918. doi: 10.1038/s41598-021-90331-2 (PMC8149411; doi:10.1038/s41598-021-90331-2)
Supplement: Supplementary file 5 — Supplementary Information. [file 41598_2021_90331_MOESM5_ESM.docx]

# Supplementary Material

**Title:**

Lidocaine and Bupivacaine as part of multimodal pain management in a C57BL/6J laparotomy mouse model.

**Authors:** Mattea S Durst^1*^, Margarete Arras^1^, Rupert Palme^2^, Steven R Talbot^3^, Paulin Jirkof^1,4^

In the supplemental material, exact p-values for significant results from Bonferroni corrected *post hoc* comparison of treatment groups and Dunnett’s *post hoc* test comparing baseline with post-procedure measurements can be found. Additionally, graphs and results excluded from the main manuscript are displayed.

## Water intake

Supplementary Table S1: Absolute values of water intake in ml per 24 h in each sex and treatment group.

| **Time** | **Contrasts** | **Male** | | **Female** | |
| --- | --- | --- | --- | --- | --- |
|  |  | **Mean (ml/24 h)** | **SD** | **Mean (ml/24 h)** | **SD** |
| **Baseline** | **PaLiBupOP** | 5.33 | 1.50 | 5.42 | 1.06 |
|  | **LiBupOP** | 5.72 | 1.75 | 5.18 | 0.814 |
|  | **PaOP** | 5.41 | 1.36 | 5.39 | 1.25 |
|  | **PaAn** | 5.18 | 1.08 | 5.52 | 1.09 |
|  | **Pa** | 5.95 | 0.799 | 7.04 | 0.374 |
| **24 h post-procedure** | **PaLiBupOP** | 3.92 | 1.91 | 3.54 | 1.95 |
|  | **LiBupOP** | 4.55 | 1.29 | 4.76 | 1.20 |
|  | **PaOP** | 4.51 | 1.48 | 3.42 | 1.98 |
|  | **PaAn** | 3.72 | 2.14 | 4.04 | 1.75 |
|  | **Pa** | 6.53 | 1.06 | 5.49 | 0.669 |
| **48 h post-procedure** | **PaLiBupOP** | 4.83 | 1.41 | 5.45 | 1.58 |
|  | **LiBupOP** | 5.16 | 0.711 | 5.28 | 0.739 |
|  | **PaOP** | 4.75 | 1.41 | 5.14 | 1.93 |
|  | **PaAn** | 5.23 | 1.09 | 5.15 | 1.69 |
|  | **Pa** | 4.76 | 0.559 | 5.30 | 0.828 |

Supplementary Table S2: Bonferroni corrected *post hoc* comparison of treatment groups for each sex and measurement time point of water intake.

| **Time** | **Contrasts** | **Male** | | | | **Female** | | | |
| --- | --- | --- | --- | --- | --- | --- | --- | --- | --- |
|  |  | **β** | **SE** | **t-value** | **p-value** | **β** | **SE** | **t-value** | **p-value** |
| **Baseline** | **LiBupOP – Pa** | - | - | - | - | -1.857 | 0.599 | -3.101 | 0.0216 |
| **24 h post-procedure** | **PaLiBupOP - Pa** | -2.6115 | 0.549 | -4.758 | <0.0001 | -1.947 | 0.599 | -3.252 | 0.0131 |
|  | **LiBupOP – Pa** | -1.9815 | 0.549 | -3.610 | 0.0037 | 1.340 | 0.453 | 2.960 | 0.0338 |
|  | **PaOP – Pa** | -2.0160 | 0.549 | -3.673 | 0.0029 | -2.071 | 0.599 | -3.459 | 0.0064 |
|  | **PaAn – Pa** | -2.8146 | 0.554 | -5.083 | <0.0001 | - | - | - | - |

Supplementary Table S3: Dunnett’s *post hoc* test of water intake comparing baseline with their respective post-procedure measurement within each treatment group.

|  | **Contrast:**  **Baseline to** | **Male** | | | | **Female** | | | |
| --- | --- | --- | --- | --- | --- | --- | --- | --- | --- |
| **Treatment** |  | **β** | **SE** | **t-value** | **p-value** | **β** | **SE** | **t-value** | **p-value** |
| **PaLiBupOP** | **24 h post-procedure** | -1.4112 | 0.452 | -3.120 | 0.0213 | -1.879 | 0.428 | -4.389 | 0.0002 |
| **PaOP** | **24 h post-procedure** | - | - | - | - | -1.972 | 0.428 | -4.608 | 0.0001 |
| **PaAn** | **24 h post-procedure** | -1.4692 | 0.465 | -3.162 | 0.0185 | -1.475 | 0.428 | -3.446 | 0.0072 |

## Body weight

Supplementary Table S4: Bonferroni corrected *post hoc* comparison of treatment groups for each sex and measurement time point of body weight.

| **Time** | **Contrasts** | **Male** | | | |
| --- | --- | --- | --- | --- | --- |
|  |  | **β** | **SE** | **t-value** | **p-value** |
| **24 h post-procedure** | **PaOP - Pa** | -2.3400 | 0.781 | -2.996 | 0.0343 |

Supplementary Table S5: Dunnett’s *post hoc* test of body weight comparing baseline with respective post-procedure measurement within each treatment group.

|  | **Contrast:**  **PreOP to** | **Male** | | | | **Female** | | | |
| --- | --- | --- | --- | --- | --- | --- | --- | --- | --- |
| **Treatment** |  | **β** | **SE** | **t-value** | **p-value** | **β** | **SE** | **t-value** | **p-value** |
| **PaLiBupOP** | **24 h post-procedure** | -1.116 | 0.237 | -4.711 | 0.0001 | -1.249 | 0.259 | -4.815 | <0.0001 |
|  | **48 h post-procedure** | -0.722 | 0.237 | -3.046 | 0.0269 | - | - | - | - |
| **PaOP** | **24 h post-procedure** | -1.338 | 0.237 | -5.648 | <0.0001 | -0.7625 | 0.259 | -2.941 | 0.0374 |
|  | **48 h post-procedure** | -1.240 | 0.237 | -5.234 | <0.0001 | - | - | - | - |
| **PaAn** | **24 h post-procedure** | -0.952 | 0.237 | -4.017 | 0.0009 | - | - | - | - |

## Food intake

Food intake was measured by weighing food pellets every 24 h for animals in setup 1. The intake is shown in grams per 24 h for each group 24 h after surgery/anesthesia (24 h post-procedure) and the second 24 h after surgery/anesthesia (48 h post-procedure) in Supplementary Figure S1.

Supplementary Table S6: Bonferroni corrected *post hoc* comparison of treatment groups for food intake.

| **Time** | **Contrasts** | **Male** | | | | **Female** | | | |
| --- | --- | --- | --- | --- | --- | --- | --- | --- | --- |
|  |  | **β** | **SE** | **t-value** | **p-value** | **β** | **SE** | **t-value** | **p-value** |
| **24 h post-procedure** | **PaLiBupOP - LiBupOP** | -1.3630 | 0.360 | -3.781 | 0.0016 | -1.505 | 0.396 | -3.802 | 0.0014 |
|  | **LiBupOP - PaOP** | 1.0260 | 0.360 | 2.846 | 0.0321 | - | - | - | - |
| **48 h post-procedure** | **LiBupOP - PaOP** | 1.0840 | 0.360 | 3.007 | 0.0199 | - | - | - | - |

Supplementary Table S7: Dunnett’s *post hoc* test of food intake comparing baseline with their respective post-procedure measurement within each treatment group.

|  | **Contrast:**  **Baseline to** | **Male** | | | | **Female** | | | |
| --- | --- | --- | --- | --- | --- | --- | --- | --- | --- |
| **Treatment** |  | **β** | **SE** | **t-value** | **p-value** | **β** | **SE** | **t-value** | **p-value** |
| **PaLiBupOP** | **24 h post-procedure** | -1.922 | 0.364 | -5.273 | <0.0001 | -2.212 | 0.396 | -5.589 | <0.0001 |
|  | **48 h post-procedure** | -1.383 | 0.364 | -3.794 | 0.0025 | -1.353 | 0.396 | -3.418 | 0.0083 |
| **PaOP** | **24 h post-procedure** | -1.093 | 0.377 | -2.897 | 0.0399 | -1.514 | 0.396 | -3.825 | 0.0022 |
|  | **48 h post-procedure** | -1.207 | 0.377 | -3.200 | 0.0164 | -1.418 | 0.396 | -3.583 | 0.0049 |
| **PaAn** | **24 h post-procedure** | -1.172 | 0.377 | -3.109 | 0.0216 | - | - | - | - |
|  | **48 h post-procedure** | -1.219 | 0.377 | -3.233 | 0.0148 | - | - | - | - |

## Mouse Grimace Scale

Supplementary Table S8: Bonferroni corrected *post hoc* comparison of treatment groups for each sex and measurement time point of the Mouse Grimace Scale.

| **Time** | **Contrasts** | **Male** | | | | **Female** | | | |
| --- | --- | --- | --- | --- | --- | --- | --- | --- | --- |
|  |  | **β** | **SE** | **t-value** | **p-value** | **β** | **SE** | **t-value** | **p-value** |
| **1 h post-procedure** | **PaLiBupOP - LiBupOP** | 0.161 | 0.0537 | 2.997 | 0.0302 | 0.327 | 0.0616 | 5.310 | <0.0001 |
|  | **PaLiBupOP - Pa** | - | - | - | - | 0.234 | 0.0576 | 4.070 | 0.0006 |
|  | **LiBupOP - PaOP** | -0.204 | 0.0498 | -4.091 | 0.0006 | -0.249 | 0.0617 | -4.039 | 0.0007 |
|  | **LiBupOP - PaAn** | -0.258 | 0.0498 | -5.179 | <0.0001 | -0.269 | 0.0616 | -4.357 | 0.0002 |
|  | **LiBupOP - Pa** | -0.152 | 0.0508 | -2.989 | 0.0312 | - | - | - | - |
|  | **PaAn - Pa** | - | - | - | - | 0.176 | 0.0576 | 3.049 | 0.0254 |
| **3 h post-procedure** | **PaLiBupOP - LiBupOP** | 0.273 | 0.0487 | 5.596 | <0.0001 | 0.294 | 0.0530 | 5.536 | <0.0001 |
|  | **LiBupOP - PaOP** | -0.302 | 0.0487 | -6.209 | <0.0001 | -0.293 | 0.0530 | -5.534 | <0.0001 |
|  | **LiBupOP - PaAn** | -0.338 | 0.0487 | -6.941 | <0.0001 | -0.312 | 0.0530 | -5.884 | <0.0001 |
|  | **LiBupOP - Pa** | -0.204 | 0.0487 | -4.189 | 0.0004 | -0.186 | 0.0548 | -3.388 | 0.0082 |
| **6 h post-procedure** | **PaLiBupOP - LiBupOP** | 0.3160 | 0.0508 | 6.223 | <0.0001 | 0.398 | 0.0543 | 7.329 | <0.0001 |
|  | **LiBupOP - PaOP** | -0.381 | 0.0498 | -7.650 | <0.0001 | -0.451 | 0.0543 | -8.306 | <0.0001 |
|  | **LiBupOP - PaAn** | -0.382 | 0.0498 | -7.684 | <0.0001 | -0.386 | 0.0530 | -7.281 | <0.0001 |
|  | **LiBupOP - Pa** | -0.303 | 0.0498 | -6.093 | <0.0001 | -0.332 | 0.0561 | -5.920 | <0.0001 |
| **24 h post-procedure** | **PaLiBupOP - LiBupOP** | 0.148 | 0.0487 | 3.043 | 0.0265 | 0.289 | 0.0530 | 5.443 | <0.0001 |
|  | **LiBupOP - PaOP** | -0.171 | 0.0487 | -3.512 | 0.0055 | -0.328 | 0.0530 | -6.187 | <0.0001 |
|  | **LiBupOP - PaAn** | -0.262 | 0.0487 | -5.377 | <0.0001 | -0.285 | 0.0517 | -5.516 | <0.0001 |
|  | **LiBupOP - Pa** | -0.147 | 0.0487 | -3.026 | 0.0280 | -0.297 | 0.0548 | -5.412 | <0.0001 |

Supplementary Table S9: Dunnett’s *post hoc* test of the Mouse Grimace Scale comparing baseline with their respective post-procedure measurements within each treatment group.

|  | **Contrast** | **Male** | | | | **Female** | | | |
| --- | --- | --- | --- | --- | --- | --- | --- | --- | --- |
| **Treatment** |  | **β** | **SE** | **t-value** | **p-value** | **β** | **SE** | **t-value** | **p-value** |
| **PaLiBupOP** | **B 1h – 1 h post-procedure** | 0.3873 | 0.0464 | 8.348 | <0.0001 | 0.5043 | 0.0504 | 10.009 | <0.0001 |
| **LiBupOP** |  | 0.2183 | 0.0421 | 5.180 | <0.0001 | 0.0188 | 0.0566 | 3.334 | 0.0297 |
| **PaOP** |  | 0.4902 | 0.0421 | 11.641 | <0.0001 | 0.4551 | 0.0515 | 8.839 | <0.0001 |
| **PaAn** |  | 0.5042 | 0.0421 | 11.972 | <0.0001 | 0.4696 | 0.0518 | 9.070 | <0.0001 |
| **Pa** |  | 0.5003 | 0.0421 | 11.879 | <0.0001 | 0.3177 | 0.0528 | 6.016 | <0.0001 |
| **PaLiBupOP** | **B 3h – 3 h post-procedure** | 0.4775 | 0.0409 | 11.688 | <0.0001 | 0.4686 | 0.0487 | 9.629 | <0.0001 |
| **LiBupOP** |  | 0.1925 | 0.0409 | 4.712 | 0.0001 | 0.1935 | 0.0472 | 4.096 | 0.0017 |
| **PaOP** |  | 0.5163 | 0.0409 | 12.638 | <0.0001 | 0.4395 | 0.0501 | 8.775 | <0.0001 |
| **PaAn** |  | 0.5067 | 0.0409 | 12.401 | <0.0001 | 0.4812 | 0.0487 | 9.889 | <0.0001 |
| **Pa** |  | 0.4740 | 0.0409 | 11.602 | <0.0001 | 0.3483 | 0.0528 | 6.595 | <0.0001 |
| **PaLiBupOP** | **B 6h – 6 h post-procedure** | 0.4235 | 0.0422 | 10.046 | <0.0001 | 0.4215 | 0.0487 | 8.660 | <0.0001 |
| **PaOP** |  | 0.4318 | 0.0409 | 10.570 | <0.0001 | 0.4435 | 0.0501 | 8.849 | <0.0001 |
| **PaAn** |  | 0.4975 | 0.0409 | 12.177 | <0.0001 | 0.3897 | 0.0472 | 8.249 | <0.0001 |
| **Pa** |  | 0.4223 | 0.0409 | 10.337 | <0.0001 | 0.3760 | 0.0528 | 7.120 | <0.0001 |
| **PaLiBupOP** | **B 1h – 24 h post-procedure** | - | - | - | - | 0.2737 | 0.0487 | 5.624 | <0.0001 |
| **PaOP** |  | 0.1917 | 0.0421 | 4.552 | 0.0002 | 0.3422 | 0.0501 | 6.827 | <0.0001 |
| **PaAn** |  | 0.2423 | 0.0421 | 5.755 | <0.0001 | 0.2943 | 0.0487 | 6.049 | <0.0001 |
| **Pa** |  | 0.2298 | 0.0409 | 5.626 | <0.0001 | 0.3296 | 0.0528 | 6.241 | <0.0001 |

## Von Frey Test

The von Frey test was conducted to assess hypersensitivity around the surgical wound area for animals in setup 2. The score is displayed for the baseline and post-procedure values in Supplementary Figure S2.

Supplementary Table S10: Bonferroni corrected *post hoc* comparison of treatment groups for each sex and measurement time point for the von Frey score.

| **Time** | **Contrasts** | **Male** | | | |
| --- | --- | --- | --- | --- | --- |
|  |  | **β** | **SE** | **t-value** | **p-value** |
| **Post-procedure** | **PaLiBupOP - Pa** | 3.3 | 1.06 | 3.112 | 0.0261 |
|  | **PaOP - Pa** | 3.3 | 1.06 | 3.112 | 0.0261 |

Supplementary Table S11: Dunnett’s *post hoc* test of von Frey score comparing baseline with their respective post-procedure measurement within each treatment group.

|  | **Contrast:**  **Baseline to** | **Male** | | | |
| --- | --- | --- | --- | --- | --- |
| **Treatment** |  | **β** | **SE** | **t-value** | **p-value** |
| **PaLiBupOP** | **24 h post-procedure** | 2.2 | 0.806 | 2.730 | 0.0450 |
| **PaOP** | **24 h post-procedure** | 2.7 | 0.806 | 3.351 | 0.0082 |

## Distance Moved

Distance moved of animals in setup 1 was measured during 24 h before and after surgery/anesthesia to assess activity (see Supplementary Figure S3). Due to technical issues, power failure and defective hard drives, we were unable to generate results for each animal. The baseline distance moved of over 3000 m by one female animal was excluded from the analysis as we suspected a technical error in this case.

Supplementary Table S12: Bonferroni corrected *post hoc* comparison of treatment groups for moved distance.

| **Time** | **Contrasts** | **Male** | | | | **Female** | | | |
| --- | --- | --- | --- | --- | --- | --- | --- | --- | --- |
|  |  | **β** | **SE** | **t-value** | **p-value** | **β** | **SE** | **t-value** | **p-value** |
| **24 h post-procedure** | **LiBupOP - PaOP** | 215.32 | 77.5 | 2.780 | 0.0449 | - | - | - | - |

Supplementary Table S13: Dunnett’s *post hoc* test of moved distance comparing baseline with their respective post-procedure measurement within each treatment group.

|  | **Contrast:**  **Baseline to** | **Male** | | | | **Female** | | | |
| --- | --- | --- | --- | --- | --- | --- | --- | --- | --- |
| **Treatment** |  | **β** | **SE** | **t-value** | **p-value** | **β** | **SE** | **t-value** | **p-value** |
| **PaLiBupOP** | **24 h post-procedure** | -276 | 54.0 | -5.113 | 0.0001 | -261 | 68.2 | -3.818 | 0.0027 |
| **LiBupOP** | **24 h post-procedure** | -193 | 54.0 | -3.579 | 0.0053 | -312 | 67.4 | -4.630 | 0.0003 |
| **PaOP** | **24 h post-procedure** | -222 | 57.1 | -3.881 | 0.0022 | -207 | 71.0 | -2.910 | 0.0263 |
| **PaAn** | **24 h post-procedure** | -152 | 54.0 | -2.814 | 0.0360 | -191 | 67.4 | -2.829 | 0.0331 |

## Burrowing Behavior

Supplementary Table S14: Bonferroni corrected *post hoc* comparison of treatment groups for burrowing behavior.

| **Time** | **Contrasts** | **Male** | | | | **Female** | | | |
| --- | --- | --- | --- | --- | --- | --- | --- | --- | --- |
|  |  | **β** | **SE** | **t-value** | **p-value** | **β** | **SE** | **t-value** | **p-value** |
| **B 20** | **LiBupOP - Pa** | 54.736 | 15.5 | 3.538 | 0.0048 | 53.620 | 15.0 | 3.569 | 0.0044 |
|  | **PaOP - Pa** | - | - | - | - | 53.146 | 15.0 | 3.537 | 0.0049 |
|  | **PaAn - Pa** | - | - | - | - | 52.218 | 15.0 | 3.476 | 0.0061 |
| **PP 20** | **PaLiBupOP - LiBupOP** | - | - | - | - | 73.638 | 14.2 | 5.198 | <0.0001 |
|  | **PaLiBupOP - Pa** | 73.679 | 15.5 | 4.762 | <0.0001 | 67.284 | 15.0 | 4.478 | 0.0001 |
|  | **LiBupOP - PaOP** | - | - | - | - | -63.756 | 14.2 | -4.501 | 0.0001 |
|  | **LiBupOP - PaAn** | - | - | - | - | -40.899 | 14.2 | -2.887 | 0.0427 |
|  | **LiBupOP - Pa** | 75.420 | 15.5 | 4.875 | <0.0001 | - | - | - | - |
|  | **PaOP - PaAn** | 62.421 | 15.5 | 4.035 | 0.0007 | - | - | - | - |
|  | **PaOP - Pa** | 97.965 | 15.5 | 6.332 | <0.0001 | 57.401 | 15.0 | 3.821 | 0.0017 |
| **PP 8** | **PaLiBupOP - LiBupOP** | - | - | - | - | 41.806 | 14.2 | 2.951 | 0.0350 |
|  | **PaLiBupOP - Pa** | - | - | - | - | 43.927 | 15.0 | 2.924 | 0.0382 |

Supplementary Table S15: Dunnett’s *post hoc* test of burrowing behavior comparing baseline with their respective post-procedure measurements within each treatment group.

|  | **Contrast** | **Male** | | | | **Female** | | | |
| --- | --- | --- | --- | --- | --- | --- | --- | --- | --- |
| **Treatment** |  | **β** | **SE** | **t-value** | **p-value** | **β** | **SE** | **t-value** | **p-value** |
| **PaLiBupOP** | **B 20 – PP 20** | - | - | - | - | 47.26 | 12.8 | 3.686 | 0.0072 |
| **PaOP** |  | 64.64 | 15.2 | 4.246 | 0.0008 | - | - | - | - |

## Nest Building Behavior

Supplementary Table S16: Bonferroni corrected *post hoc* comparison of treatment groups for each sex and measurement time point of nest complexity score.

| **Time** | **Contrasts** | **Male** | | | | **Female** | | | |
| --- | --- | --- | --- | --- | --- | --- | --- | --- | --- |
|  |  | **β** | **SE** | **t-value** | **p-value** | **β** | **SE** | **t-value** | **p-value** |
| **6 h post-procedure** | **PaLiBupOP - LiBupOP** | -1.6 | 0.505 | -3.168 | 0.0108 | - | - | - | - |
|  | **LiBupOP - PaOP** | 1.4 | 0.505 | 2.772 | 0.0369 | - | - | - | - |
| **12 h post-procedure** | **PaLiBupOP - LiBupOP** | -1.8 | 0.505 | -3.565 | 0.0028 | - | - | - | - |
|  | **LiBupOP - PaOP** | 1.5 | 0.505 | 2.970 | 0.0203 | - | - | - | - |
| **24 h post-procedure** | **PaLiBupOP - LiBupOP** | -2.6 | 0.505 | -5.149 | <0.0001 | -1.7 | 0.406 | -4.188 | 0.0003 |
|  | **LiBupOP - PaOP** | 2.5 | 0.505 | 4.951 | <0.0001 | 1.2 | 0.406 | 2.956 | 0.0215 |
|  | **LiBupOP - PaAn** | 2.2 | 0.505 | 4.357 | 0.0001 | 1.8 | 0.406 | 4.434 | 0.0001 |

Supplementary Table S17: Dunnett’s *post hoc* test of nest complexity score comparing baseline with their respective post-procedure measurements within each treatment group.

|  | **Contrast** | **Male** | | | | **Female** | | | |
| --- | --- | --- | --- | --- | --- | --- | --- | --- | --- |
| **Treatment** |  | **β** | **SE** | **t-value** | **p-value** | **β** | **SE** | **t-value** | **p-value** |
| **PaLiBupOP** | **B 3h – 3 h post-procedure** | - | - | - | - | -1.7 | 0.333 | -5.113 | <0.0001 |
| **LiBupOP** |  | -1.5 | 0.426 | -3.524 | 0.0141 | -1.8 | 0.333 | -5.413 | <0.0001 |
| **PaOP** |  | - | - | - | - | -1.4 | 0.333 | -4.210 | 0.0010 |
| **PaAn** |  | -1.5 | 0.426 | -3.524 | 0.0141 | -1.3 | 0.333 | -3.910 | 0.0033 |
| **PaLiBupOP** | **B 6h – 6 h post-procedure** | -1.8 | 0.426 | -4.229 | 0.0009 | -2.1 | 0.333 | -6.316 | <0.0001 |
| **LiBupOP** |  | - | - | - | - | -1.3 | 0.333 | -3.910 | 0.0033 |
| **PaOP** |  | -1.6 | 0.426 | -3.759 | 0.0059 | -1.5 | 0.333 | -4.511 | 0.0033 |
| **PaAn** |  | -2.1 | 0.426 | -4.934 | <0.0001 | -1.8 | 0.333 | -5.413 | <0.0001 |
| **PaLiBupOP** | **B 12h – 12 h post-procedure** | -1.9 | 0.426 | -4.464 | 0.0003 | -2.0 | 0.333 | -6.015 | <0.0001 |
| **LiBupOP** |  | - | - | - | - | -1.5 | 0.333 | -4.511 | 0.0003 |
| **PaOP** |  | -1.8 | 0.426 | -4.229 | 0.0009 | -1.7 | 0.333 | -5.113 | <0.0001 |
| **PaAn** |  | -2.3 | 0.426 | -5.404 | <0.0001 | -1.8 | 0.333 | -5.413 | <0.0001 |
| **PaLiBupOP** | **B – 24 h post-procedure** | -1.5 | 0.426 | -3.524 | 0.0141 | - | - | - | - |
| **LiBupOP** |  | - | - | - | - | 1.7 | 0.333 | 5.113 | <0.0001 |
| **PaOP** |  | -2.0 | 0.426 | -4.699 | 0.0001 | - | - | - | - |
| **PaAn** |  | -1.8 | 0.426 | -4.229 | 0.0009 | - | - | - | - |

## Fecal Corticosterone Metabolites

Supplementary Table S18: Bonferroni corrected *post hoc* comparison of treatment groups for each sex and measurement time point of fecal corticosterone metabolites.

| **Time** | **Contrasts** | **Male** | | | | **Female** | | | |
| --- | --- | --- | --- | --- | --- | --- | --- | --- | --- |
|  |  | **β** | **SE** | **t-value** | **p-value** | **β** | **SE** | **t-value** | **p-value** |
| **1 h post-procedure** | **PaLiBupOP - LiBupOP** | - | - | - | - | 62.14 | 22.1 | 2.809 | 0.0332 |
| **6 h post-procedure** | **PaLiBupOP - LiBupOP** | 45.450 | 14.3 | 3.167 | 0.0108 | 63.86 | 23.2 | 2.752 | 0.0390 |
| **24 h post-procedure** | **PaLiBupOP - LiBupOP** | - | - | - | - | 138.52 | 22.1 | 6.266 | <0.0001 |
|  | **PaLiBupOP - PaOP** | - | - | - | - | 64.53 | 23.2 | 2.781 | 0.0358 |
|  | **PaLiBupOP - PaAn** | - | - | - | - | 95.73 | 22.6 | 4.238 | 0.0002 |
|  | **LiBupOP - PaOP** | - | - | - | - | -73.99 | 22.7 | -3.256 | 0.0081 |

Supplementary Table S19: Dunnett’s *post hoc* test of fecal corticosterone metabolites comparing baseline with their respective post-procedure measurements within each treatment group.

|  | **Contrast** | **Male** | | | | **Female** | | | |
| --- | --- | --- | --- | --- | --- | --- | --- | --- | --- |
| **Treatment** |  | **β** | **SE** | **t-value** | **p-value** | **β** | **SE** | **t-value** | **p-value** |
| **PaLiBupOP** | **B 1h – 1 h post-procedure** | - | - | - | - | 78.34 | 19.3 | 4.064 | 0.0017 |
| **PaLiBupOP** | **B 3h – 3 h post-procedure** | - | - | - | - | 89.52 | 20.8 | 4.299 | 0.0006 |
| **LiBupOP** |  | 40.99 | 11.08 | 3.698 | 0.0072 | - | - | - | - |
| **PaOP** |  | - | - | - | - | 69.25 | 20.0 | 3.467 | 0.0155 |
| **PaAn** |  | - | - | - | - | 65.38 | 18.7 | 3.499 | 0.0139 |
| **PaLiBupOP** | **B 6h – 6 h post-procedure** | 83.96 | 13.36 | 6.283 | <0.0001 | 95.39 | 20.0 | 4.778 | 0.0001 |
| **LiBupOP** |  | 43.59 | 10.83 | 4.027 | 0.0021 | 72.83 | 19.3 | 3.780 | 0.0050 |
| **PaOP** |  | 69.50 | 13.00 | 5.345 | <0.0001 | 76.68 | 20.8 | 3.780 | 0.0071 |
| **PaAn** |  | 50.72 | 10.99 | 4.617 | 0.0003 | 90.74 | 20.0 | 4.547 | 0.0002 |
| **PaLiBupOP** | **B 1h – 24 h post-procedure** | - | - | - | - | 147.98 | 19.3 | 7.684 | <0.0001 |
| **PaOP** |  | - | - | - | - | 72.22 | 20.4 | 3.545 | 0.0118 |
| **PaAn** |  | 33.90 | 9.74 | 3.479 | 0.0158 | - | - | - | - |

## Sugar Consumption

Sugar consumption was measured before and on the two days following surgery/anesthesia to test for anhedonia in the animals of setup 2.

Supplementary Table S20: Bonferroni corrected *post hoc* comparison of treatment groups for each sex and measurement time point of sugar intake.

| **Time** | **Contrasts** | **Male** | | | |
| --- | --- | --- | --- | --- | --- |
|  |  | **β** | **SE** | **t-value** | **p-value** |
| **Baseline** | **PaLiBupOP - Pa** | 0.132 | 0.0394 | 3.346 | 0.0125 |
|  | **LiBupOP - Pa** | 0.129 | 0.0394 | 3.270 | 0.0158 |
| **48 h post-procedure** | **PaLiBupOP - Pa** | 0.131 | 0.0394 | 3.321 | 0.0135 |
|  | **LiBupOP - Pa** | 0.125 | 0.0394 | 3.169 | 0.0216 |

Supplementary Table S21: Dunnett’s *post hoc* test of sugar intake comparing baseline with post-procedure measurements within each treatment group.

|  | **Contrast** | **Male** | | | |
| --- | --- | --- | --- | --- | --- |
| **Treatment** |  | **β** | **SE** | **t-value** | **p-value** |
| **PaOP** | **Baseline – 24 h post-procedure** | -0.090 | 0.0255 | -3.525 | 0.0067 |

## Figure legends

Supplementary Figure S1: **Food intake** in grams per 24 h at baseline, for the first 24 h (24 h post-procedure) and the second 24 h after the procedure (48 h post-procedure). Pairwise comparison with Bonferroni correction detected no significant difference between the treatment groups in the baseline food intake in both sexes. Within male mice, food intake in LiBupOP was significantly higher than in PaLiBupOP and PaOP 24 h post-procedure. 48 h post-procedure food intake in LiBupOP was significantly higher than in PaOP. In female mice, significantly higher food intake was detected 24 h post-procedure in LiBupOP compared to PaLiBupOP. Significant differences from the respective baseline measurements within each treatment group are indicated with *, p≤0.05, Dunnett’s *post hoc* test. Data are shown as scatter dot plot with mean ± SD. Numbers per group: PaLiBupOP, LiBupOP, PaOP, and PaAn *n*=10/per sex.

Figure S3: **Distance moved** in meter per 24 h displayed for each treatment group in male and female mice. In both sexes, Bonferroni *post hoc* comparison detected no differences between treatment groups at baseline level. In male mice, LiBupOP moved a significantly greater distance than PaOP in the 24 h post-procedure. Significant differences to baseline measurements are indicated with *, p≤0.05, Dunnett’s *post hoc*. Due to technical issues data could not be gathered for each animal. Numbers per group: PaLiBupOP, LiBupOP, PaOP and PaAn n=10/per sex.

Supplementary Figure S2: The **von Frey score** in male and female animals displayed for each treatment group for baseline and post-procedure time points. Bonferroni *post hoc* testing showed no significant differences between the treatment groups at baseline level. In male Pa, post-procedure von Frey score was significantly lower compared to PaLiBupOP and PaOP. Significant differences from the respective baseline measurements are indicated with *, p≤0.05, Dunnett’s *post hoc* test. Data are shown as scatter dot plot with mean ± SD. Numbers per group: PaLiBupOP, LiBupOP, PaOP and PaAn *n*=10/per sex; Pa male *n*=10, Pa female *n*=8.

Supplementary Figure S4: **Sugar intake** in female and male mice displayed for each treatment group at baseline, 24 h, and 48 h after surgery or anesthesia. *Post hoc* comparison of treatment groups with Bonferroni correction revealed that male Pa had significantly lower sugar intake than PaLiBupOP and LiBupOP at baseline level and 48 h post-procedure. The test revealed no significant differences between treatment groups in female mice at baseline and post-procedure levels. Significant differences to baseline measurements are indicated with *, p≤0.05. Data are shown as scatter dot plot with mean ± SD. Numbers per group: PaLiBupOP, LiBupOP, PaOP, and PaAn *n*=10/per sex; Pa male *n*=10, Pa female *n*=8.
